# ICOS agonism by JTX-2011 (vopratelimab) requires initial T cell priming and Fc cross-linking for optimal T cell activation and anti-tumor immunity in preclinical models

**DOI:** 10.1371/journal.pone.0239595

**Published:** 2020-09-24

**Authors:** Amanda Hanson, Kutlu Elpek, Ellen Duong, Lindsey Shallberg, Martin Fan, Calvin Johnson, Matthew Wallace, George R. Mabry, Stephen Sazinsky, Lauren Pepper, Chengyi J. Shu, Sriram Sathyanarayanan, Sarah Zuerndorfer, Tyler Simpson, Monica Gostissa, Michael Briskin, Deborah Law, Jennifer Michaelson, Christopher J. Harvey

**Affiliations:** 1 Preclinical Sciences, Jounce Therapeutics, Inc., Cambridge, Massachusetts, United States of America; 2 Protein Sciences, Jounce Therapeutics, Inc., Cambridge, Massachusetts, United States of America; 3 Translational Sciences, Jounce Therapeutics, Inc., Cambridge, Massachusetts, United States of America; 4 Pharmacology, Jounce Therapeutics, Inc., Cambridge, Massachusetts, United States of America; 5 Research, Jounce Therapeutics, Inc., Cambridge, Massachusetts, United States of America; University of Michigan Medical School, UNITED STATES

## Abstract

Immunotherapy checkpoint inhibitors, such as antibodies targeting PD-1 and CTLA-4, have demonstrated the potential of harnessing the immune system to treat cancer. However, despite encouraging results particularly with respect to survival, only a minority of patients benefit from these therapies. In clinical studies aimed at understanding changes in the immune system following immunotherapy treatment, ICOS (Inducible T cell CO-Stimulator) was shown to be significantly up-regulated on CD4+ T cells and this was associated with clinical activity, indicating that ICOS stimulatory activity may be beneficial in the treatment of solid tumors. In this report, we describe the generation of specific, species cross-reactive, agonist antibodies to ICOS, including the humanized clinical candidate, JTX-2011 (vopratelimab). Preclinical studies suggest that the ICOS stimulating antibodies require Fc receptor cross-linking for optimal agonistic activity. Notably, the ICOS antibodies do not exhibit superagonist properties but rather require T cell receptor (TCR)-mediated upregulation of ICOS for agonist activity. Treatment with the ICOS antibodies results in robust anti-tumor benefit and long-term protection in preclinical syngeneic mouse tumor models. Additional benefit is observed when the ICOS antibodies are administered in combination with anti-PD-1 and anti-CTLA-4 therapies. Based on the preclinical data, JTX-2011 is currently being developed in the clinical setting for the treatment of solid tumors.

## Introduction

The clinical success of checkpoint inhibitors in a range of cancer indications has ushered in a new era in cancer therapy. Clinical trials evaluating antibodies targeting CTLA-4 and PD-1/PD-L1 have demonstrated a significant increase in patient survival, and these treatments are rapidly becoming the new standard of care in a number of indications [1]. Given that the existing cancer immunotherapies are not effective for all patients or in all indications, there is currently great interest in generating therapeutic agents to other T cell targets, including inhibitory receptors (e.g., LAG-3 and TIM-3) and costimulatory receptors (e.g., CD137 and OX40), as either monotherapies or for use in combination with clinically approved checkpoint inhibitor antibodies [1]. As multiple molecules have been implicated as either positive or negative regulators for T cell immune responses, one of the challenges in therapeutic development is in selecting those molecules that might provide the best anti-tumor potential.

ICOS is a disulfide-linked homodimer and a member of the B7/CD28 immunoglobulin superfamily that is expressed mainly on activated T cells [2]. Its only known ligand is ICOS ligand (ICOSL; B7-H2; B7RP1; CD275), also a member of the B7 superfamily, that is expressed on B cells, macrophages and dendritic cells [2, 3]. Upon activation, ICOS induces signaling through the PI3K and AKT pathways and subsequently leads to diverse effects on T cell subsets, including proliferation, differentiation, and survival [2]. Unlike CD28, which is constitutively expressed on T cells and provides co-stimulatory signals necessary for full activation of resting T cells, ICOS is expressed only after signal 1, i.e. initial T cell priming by antigen [4]. Clinical and nonclinical data suggest that ICOS plays an important role in the immune response to cancer. Analysis of patient samples suggested a role for ICOS in the activity of anti-CTLA-4 therapy, including in melanoma patients treated with ipilumumab, where a sustained increase in the frequency of ICOS hi CD4^+^ T cells correlated with clinical benefit and improved survival [5]. These clinical translational data suggested that agonism of the ICOS pathway might be therapeutically beneficial for patients. Supportive data also comes from preclinical studies. The efficacy of CTLA-4 inhibition in melanoma bearing mice was significantly reduced in mice lacking either ICOS or its ligand [6]. Moreover, anti-tumor efficacy in pre-clinical studies observed through an ICOSL vaccination approach or recombinant oncolytic viral delivery suggests that agonism of the ICOS/ICOSL pathway can provide therapeutic benefit in the setting of cancer immunotherapy [7, 8]. Analysis of signaling pathways and mechanisms of other costimulatory molecules such as CD28, 4-1BB, CD40, OX40, and GITR also suggests that ICOS may be a common and necessary component for multiple agonist mechanisms [9–14].

To explore the potential for activation of the ICOS pathway to lead to anti-tumor immunity we generated ICOS antibodies. Here we report the characterization of a novel ICOS-specific antibody that was chosen based on in vitro and in vivo assessment of agonistic activity. A novel ICOS antibody, JTX-2011 (parental clone 37A10), was chosen based on potent stimulatory activity on CD4 T cells, including induction of proliferation, cytokine production, and AKT phosphorylation in an Fc competent format. The ICOS antibody demonstrated robust *in vivo* efficacy as both a single agent and in combination with anti-PD-1 or anti-CTLA-4 in multiple syngeneic mouse tumor models. In these models, treatment with ICOS antibody led to an increase in T effector (Teff) cells within the tumor microenvironment, together with an approximate 80% decrease in T regulatory (Treg) cells. No depletion of other T cells subsets was noted. Based on these preclinical data, the humanized ICOS antibody, JTX-2011, is currently in clinical development as a cancer immunotherapeutic.

## Results and discussion

### Generation and characterization of ICOS agonist antibodies

A panel of hamster anti-human ICOS (hICOS) antibodies was screened for binding specificity, cross-species reactivity, and biochemical and functional activity, with clone 37A10 being selected for further development. The 37A10 clone was assessed for binding to monovalent recombinant ICOS using biolayer interferometry, and for binding to cell surface expressed ICOS by flow cytometry. The affinity of 37A10 to monovalent hICOS and mouse ICOS (mICOS) was measured as 3.2 nM and 5.0 nM, respectively. Reactivity across species was further demonstrated by flow cytometry (S1A Fig in S1 File), whereby the 37A10 clone was shown to bind to CHO cells expressing hICOS (EC50: 0.083 μg/ml, 0.55 nM), cynomolgus ICOS (EC50: 0.29 μg/ml, 1.93 nM), rat ICOS (EC50: 0.087 μg/ml, 0.58 nM), and mICOS (EC50: 0.086 μg/ml, 0.57 nM), but not to the parental CHO cells.

Functional activity of 37A10 was initially assessed by an assay that employs chimeric ICOS proteins overexpressed in a reporter cell line. This assay was developed based on the finding that ICOS is generally a weak inducer of NF-κB, but an ICOS-CD28 chimera can robustly activate NF-κB and NFAT upon ligation of ICOS [15]. Consequently, we generated chimeric ICOS constructs comprised of the extracellular and transmembrane domains of hICOS or mICOS fused with the cytoplasmic domain of human CD28 or hICOS (hICOS-hCD28 and hICOS-hICOS, respectively), and overexpressed these constructs in Jurkat-NF-κB-GFP cells. The Jurkat reporter cells were stimulated with suboptimal levels of phorbol 12-myristate 13-acetate (PMA) to provide signal 1 (initial priming). The 37A10 ICOS antibody was added in the presence of sub-optimal PMA to assess its ability to provide the second, co-stimulatory signal to the T cells. Consistent with Watanabe et al [15], 37A10 induced activation of the reporter cells, as assessed by an increase in frequency of GFP expression, only in cells expressing the chimeric hICOS with the cytoplasmic domain of CD28 but not cells expressing full length ICOS (S1B Fig in S1 File). In contrast, the positive control anti-CD28 antibody stimulated both cell lines at similar levels, as anti-CD28 signals through endogenous CD28 present on the Jurkat reporter cells. Next Jurkat-hICOS-hCD28 and Jurkat-mICOS-hCD28 cells were used to compare the activity of 37A10 for human and mouse ICOS. Clone 37A10 stimulated GFP reporter activity through hICOS and mICOS in a dose-dependent manner demonstrating similar activity in both species (S1C Fig in S1 File). A hamster IgG isotype control did not have any effect over PMA alone in this assay.

Given the favorable characteristics of clone 37A10, this antibody was selected for further characterization, and a humanized IgG1 version called JTX-2011 was generated. JTX-2011 retained species cross-reactivity with affinities of 0.93 nM to hICOS, 0.46 nM to cynomolgus ICOS, 3.7 nM to rat ICOS, and 0.64 nM to mICOS. JTX-2011 activated both Jurkat-hICOS-hCD28 and Jurkat-mICOS-hCD28 cells in a dose-dependent manner, whereas an unrelated control antibody did not have any effect (S1D Fig in S1 File). Furthermore, binding studies were performed to confirm lack of reactivity to closely related CD28 family receptors, including human and mouse CD28, CTLA-4, PD-1 and BTLA (S1 Table in S1 File). This specificity of the ICOS antibodies is in contrast to the reported interactions of hICOSL with related family members, CD28 and CTLA-4, in addition to ICOS [16].

### Agonistic effects of ICOS agonist antibodies on human primary T cells

**Fig 1:**
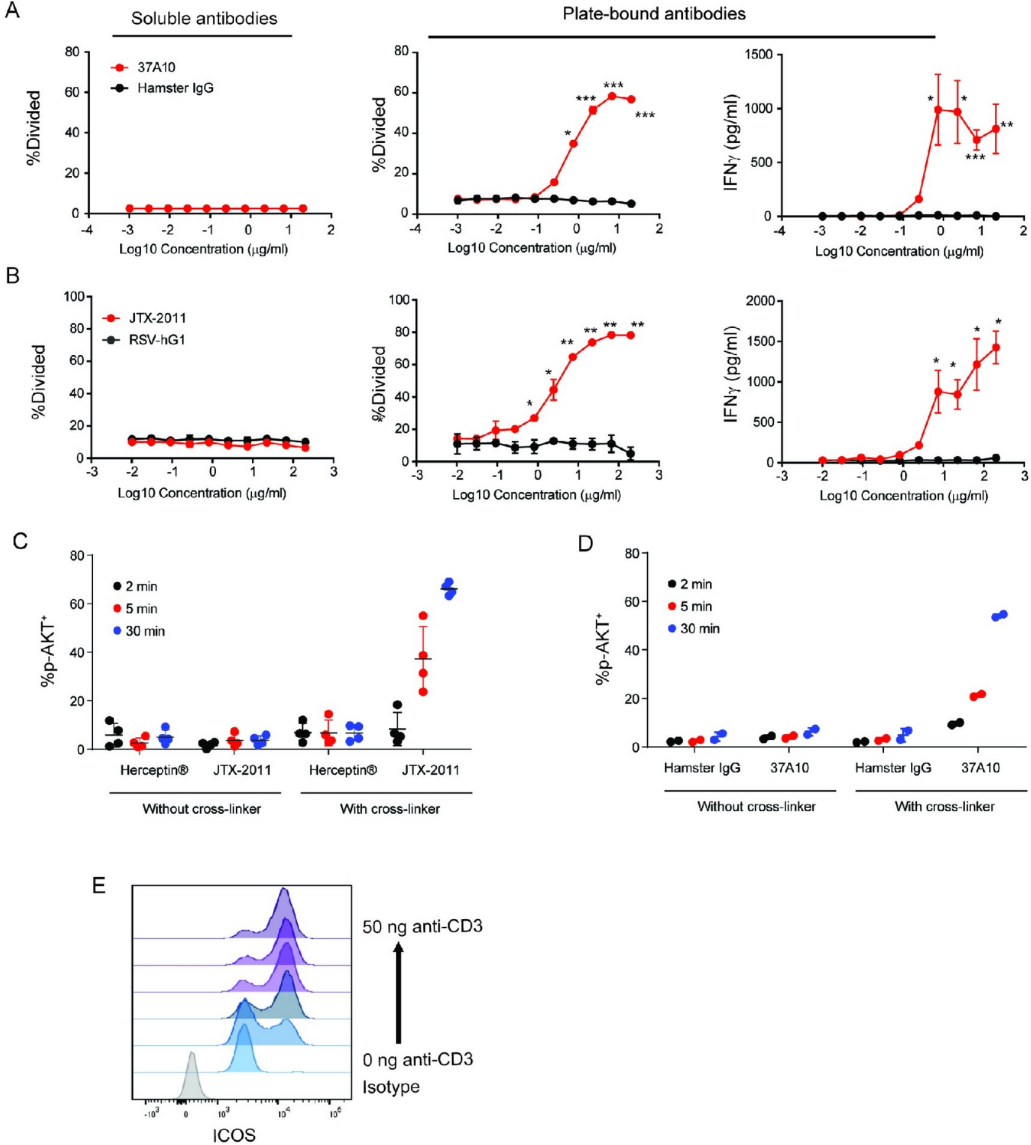
Agonistic effect of ICOS antibodies on T cells. (*A*) Purified human CD4 T cells were stimulated with serial dilutions of 37A10 or hamster IgG isotype control in soluble (left) or plate-bound (right) format in the presence of suboptimal anti-CD3 OKT3 antibody (1.0 μg/ml). Cell division was determined by CFSE dilution using flow cytometry. IFNγ production was analyzed by Cytokine Bead Arrays. (B) Repeat of A using JTX-2011 or anti-RSV-hIgG1 isotype control. (*C*) Induction of p-AKT in CD4 T cells. Cells were activated with anti-CD3/CD28 for 24 hrs, rested for 24 hrs, and then washed. Cells were incubated with JTX-2011 or Herceptin® (hIgG1 isotype control) on ice, and then incubated at 37ºC in the presence or absence of a cross-linking secondary antibody. p-AKT was measured by flow cytometry, and percentage of p-AKT^+^ cells at 2, 5 or 30 min after stimulation with JTX-2011 or Herceptin® is shown. Each symbol indicates CD4 T cells from independent donors (N = 4). (*D*) Percentage of p-AKT^+^ cells at 2, 5 or 30 min after stimulation with 37A10 or hamster IgG. Each symbol indicates CD4 T cells from independent donors (N = 2). (E) ICOS staining on CD4 T cells by flow cytometry following 24 hours of stimulation of with PBMCs with anti-CD3 antibody OKT3 at 37˚C. Data are plotted as mean ± standard deviation (SD).

We assessed the ability of 37A10 and JTX-2011 to activate primary human T cells. Purified CD4 T cells isolated from healthy donor peripheral blood mononuclear cells (PBMC) were stimulated with 37A10, JTX-2011 or isotype control antibodies for 3 days in the presence of a suboptimal concentration of plate-bound anti-CD3 antibody that was used to provide signal 1 to the T cells. While 37A10 and JTX-2011 did not have any effect when added in soluble form, both antibodies stimulated T cell proliferation in a plate-bound format in a dose-dependent manner (Fig 1A and 1B). Similar results were obtained for two other donors, and the EC50 values for the three donors were 0.11, 0.44 and 17.4 μg/ml (0.73, 2.93 and 115.54 nM, respectively) for 37A10, and 1.2, 2.8 and 6.8 μg/ml (8.00, 18.68 and 45.36 nM, respectively) for JTX-2011. Stimulation of primary T cells with plate-bound ICOS antibodies also induced IFNγ production in a dose-dependent manner (Fig 1A and 1B). Isotype control antibodies did not have any effect on T cell proliferation or cytokine production. Additional cytokine profiling from supernatants of CD4 T cells stimulated with anti-CD3 in combination with either anti-CD28 or JTX-2011 demonstrated a differential cytokine response driven by either costimulatory pathway with ICOS agonism driving IFNγ, IL-17a, IL-10, IL-9, GM-CSF, TNFα, and IL-21 secretion by polyclonally activated CD4 T cells (S2A and S2B Figs in S1 File).

As further evidence for the agonistic effect of the ICOS antibodies, we evaluated AKT phosphorylation (p-AKT), a proximal signaling event reported to be mediated by ICOS ligation [2], as measured by flow cytometry. We found that p-AKT was detected after JTX-2011 or 37A10 treatment in up to 60% of the cells by 30 min, and that this required cross-linking of the ICOS antibody, as p-AKT was only observed in the presence of a secondary cross-linking antibody (Fig 1C and 1D). An unrelated isotype control antibody, Herceptin® (Genentech, Inc.) or hamster IgG, did not have any effect.

JTX-2011 did not have any effect on ERK1/2 phosphorylation (S2C Fig in S1 File), whereas a positive control, PMA/Ionomycin, induced a rapid increase in p-ERK1/2, but not p-AKT (S2D Fig in S1 File). Previous studies showed phosphorylation of both AKT and ERK1/2 upon engagement of ICOS with ICOSL [17] or an ICOS antibody [18]. The discrepancy between these results may be due to differences in assay format or non-specific interactions with other CD28 family members as has been reported for ICOSL. Other ICOS antibodies may exhibit similar non-specific binding profiles [16]. Literature reports are consistent with our findings that signaling through ICOS leads to minimal to no activation of ERK [19–21]. Regardless of ERK activation, ICOS agonism by JTX-2011 induced robust AKT phosphorylation and downstream functional readouts such as T cell proliferation and secretion of IFNγ and other cytokines. Our data also indicated the requirement of receptor cross-linking for signaling through the ICOS pathway in these in vitro assays using purified T cell subsets. This is consistent with published literature suggesting ICOSL may be present on the cell surface as a noncovalent oligomer, supporting the hypothesis that cross-linking is necessary for optimal signaling [3].

All observed activity was dependent on pre-activation of CD4 T cells by antibody-mediated activation of TCR, suggesting the need for priming to obtain agonistic activity through ICOS. ICOS is an inducible receptor with low-level expression on resting T cells, and we hypothesized that TCR stimulation was necessary to induce ICOS expression above a threshold necessary for antibody-mediated agonism. To test this, we assessed ICOS expression on healthy donor CD4 T cells 24hrs after stimulation with a titration of anti-CD3 antibodies and observed a log-increase in ICOS MFI across all tested donors at concentrations above 50ng/mL (Fig 1E).

**Fig 2:**
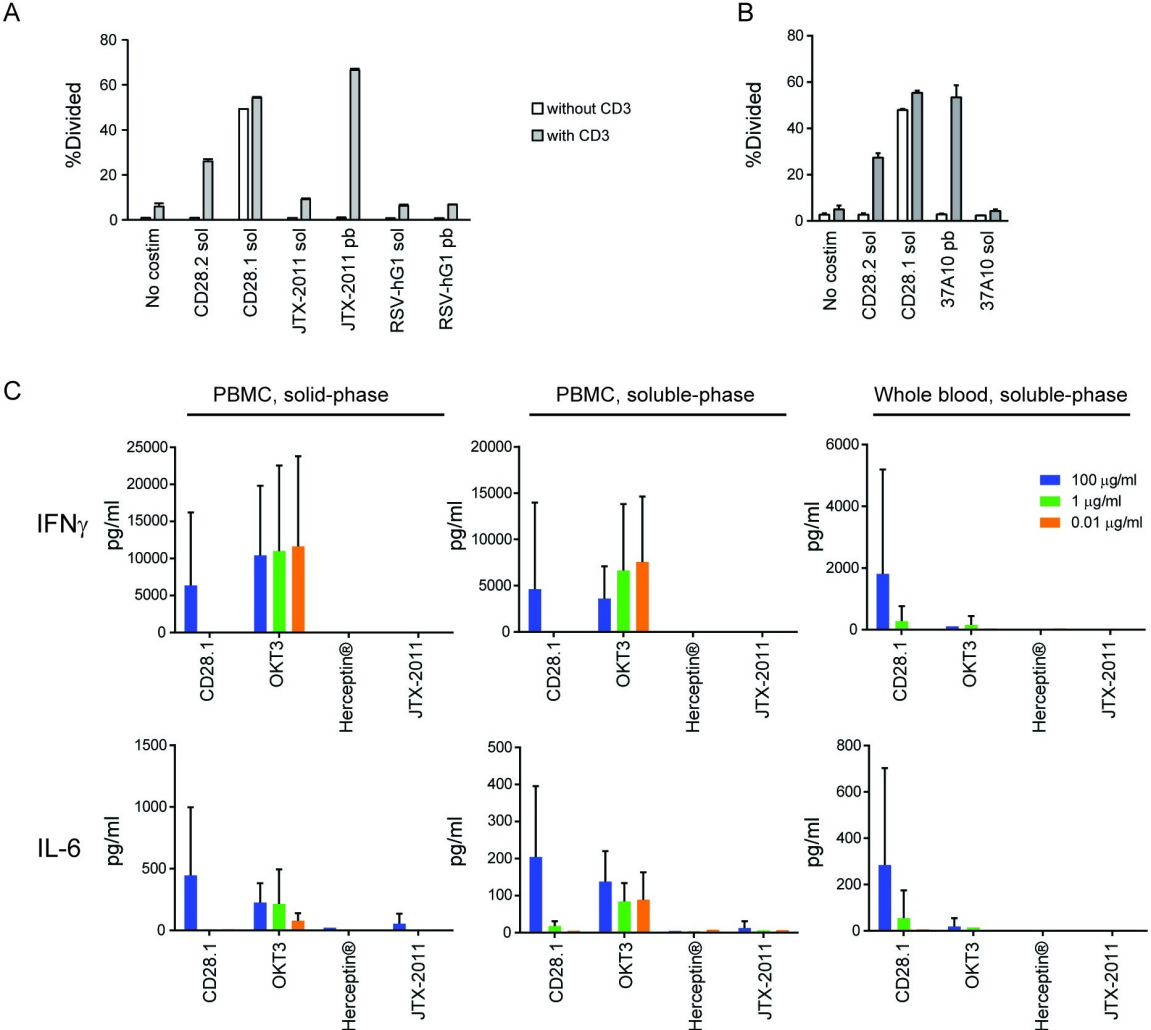
Lack of superagonism of ICOS antibodies. (*A-B*) CD4 T cells were stimulated with anti-CD28 clones in soluble form (sol), JTX-2011, 37A10 or anti-RSV-hG1 isotype control in soluble or plate-bound (pb) format at 10 μg/ml in the presence (white) or absence (gray) of anti-CD3 OKT3 antibody (1.0 μg/ml). Proliferation was measured by CFSE dilution using flow cytometry. (*C*) IFNγ (top) and IL-6 (bottom) secretion after 24 hr incubation of PBMC with CD28.1, OKT3, Herceptin® or JTX-2011 (100, 1.0 or 0.01 μg/ml; 666.7, 6.67 or 0.067 nM, respectively) in solid-phase or soluble-phase, or whole blood with antibodies in soluble-phase. Cytokines in supernatants were analyzed from a total of 6 donors for each condition. Data are plotted as mean ± SD.

Given the severe adverse effects caused in humans by the superagonist monoclonal antibody TGN1412 targeting CD28, a related family member to ICOS [22], JTX-2011 was evaluated for any potential superagonistic effects. To that end, we compared T cell proliferation mediated by JTX-2011 to two anti-CD28 clones (a superagonist and a non-superagonist) in the presence or absence of a suboptimal signal 1 provided to the T cells by anti-CD3 antibody. The previously described superagonist anti-CD28 antibody (clone CD28.1) [23], stimulated CD4 T cell proliferation either in the presence or absence of signal 1 even when added in soluble form. In contrast, even when plate-bound, JTX-2011 (Fig 2A) or parent 37A10 (Fig 2B) only induced T cell proliferation of primed T cells, similar to the effect of the non-superagonist anti-CD28 clone. These results demonstrated that the agonist ICOS antibodies did not stimulate CD4 T cell proliferation in the absence of a concurrent T cell signal 1, even in a plate-bound format.

To further investigate the superagonistic potential of JTX-2011, we performed acute cytokine release assays in three formats: PBMC activation with antibodies in solid-phase or soluble-phase and whole blood activation with antibodies in soluble-phase. Cells were treated with JTX-2011, anti-CD28 clone CD28.1 or anti-CD3 OKT3 (positive control antibodies), or Herceptin^®^ (negative control antibody [24]), and cytokines in culture supernatants were analyzed by a multiplex assay. As shown in Fig 2C (IFNɣ and IL-6) and summarized in S2 Table in S1 File (IFNɣ, IL-10, IL-13, IL-12p70, IL-1β, IL-2, IL-4, IL-6, IL-8, and TNFα), high concentrations of superagonistic CD28.1 induced secretion of the majority of the cytokines in the multiplex panel in all assay formats. OKT3 also induced all cytokines in the PBMC assays but was less potent in the whole blood format. In contrast, JTX-2011 and negative control Herceptin® did not show induction of cytokine release in any format. We also tested JTX-2011 in combination with the anti-PD-1 antibody Opdivo^®^ or the anti-CTLA-4 antibody Yervoy^®^ (Bristol-Myers Squibb Company) in the whole blood assay. Neither Opdivo^®^ nor JTX-2011 alone or in combination induced cytokine release in this assay (S3 Table in S1 File). The combination of JTX-2011 and Yervoy^®^ also resulted in no significant cytokine release in a whole blood version format (S4 Table in S1 File). Overall, these results indicated that JTX-2011 is an agonist antibody that can induce T cell proliferation, effector cytokine production and AKT phosphorylation Moreover, these data suggest that JTX-2011 does not indiscriminately activate T cells, but rather, ICOS agonist antibodies only stimulate T cells that have been previously primed, such as those recognizing tumor antigen, leading to proliferation and cytokine production by tumor-reactive T cells.

### ICOS antibody treatment as a monotherapy promotes tumor regression in the Sa1/N murine syngeneic tumor model

We next evaluated the ICOS antibodies for their potential to provide anti-tumor efficacy in murine syngeneic tumor models. To enable these studies, we generated murine Fc variants of both the parental hamster 37A10 antibody and the humanized JTX-2011 antibody to reduce the potential for immunogenicity of these antibodies in the immune-competent host mice. Specifically, 37A10-mG2a is a chimera of hamster V regions with fully functional mouse IgG2a constant regions, and likewise JTX-1011-mG2a is JTX-2011 with a mouse IgG2a constant region. These mIgG2a Fc variants were shown to activate both Jurkat-hICOS-hCD28 and Jurkat-mICOS-hCD28 reporter cells in a similar fashion to JTX-2011 (S1D Fig in S1 File).

**Fig 3:**
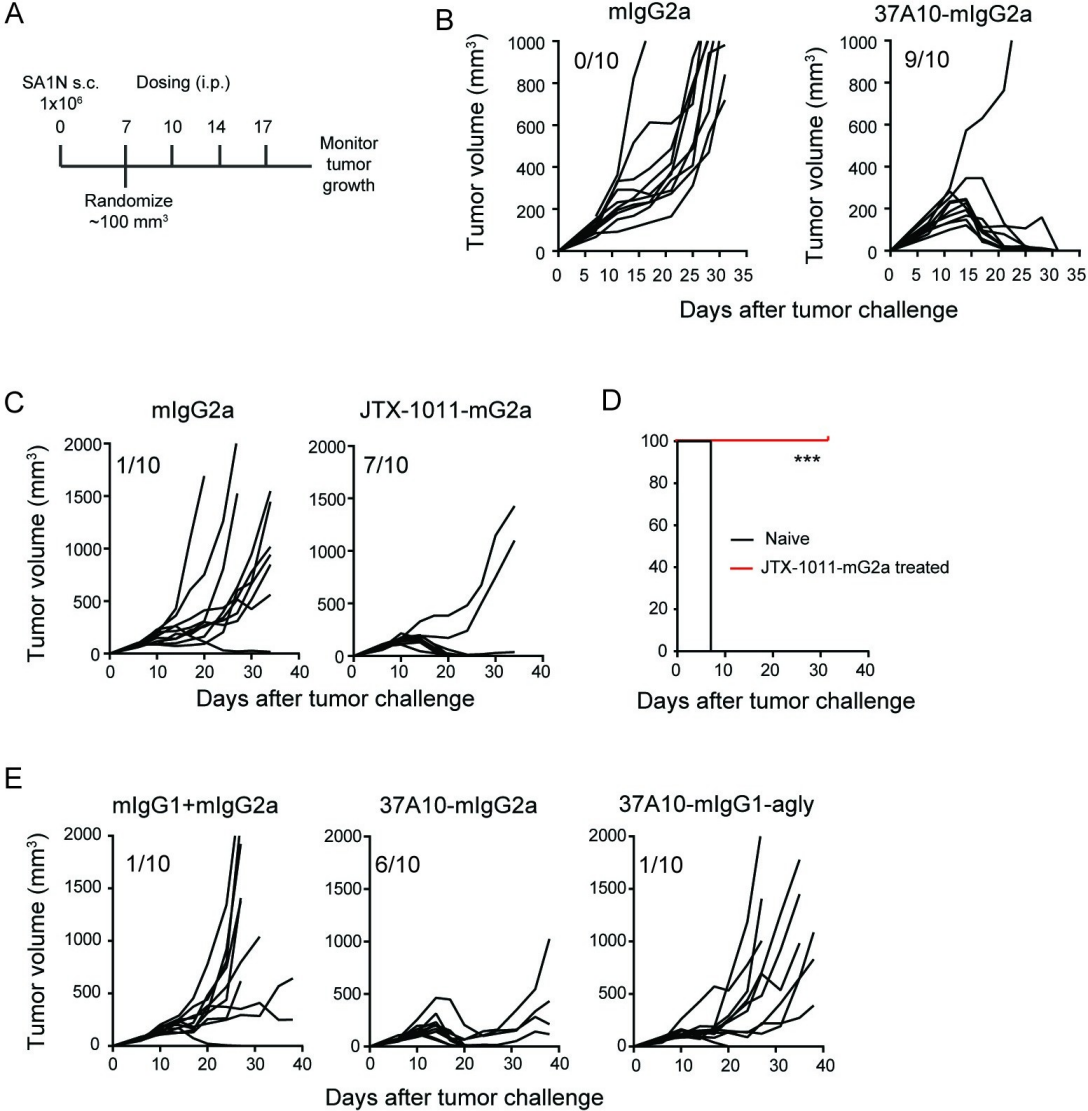
Regression of tumors and long-term protection following ICOS antibody treatment in Sa1/N model. (*A*) Dosing regimen in Sa1/N model. One million cells were injected s.c. and mice were randomized on day 7 when average tumor volume reached ~100 mm^3^. Mice received ICOS or isotype control antibodies by i.p. injections on days 7, 10, 14 and 17, and tumor growth was measured. (*B*) Tumor growth curves of mice treated with 37A10-mG2a or mIgG2a isotype (0.3 mg/kg). N = 10 per group. (*C*) Tumor growth curves of mice treated with JTX-1011-mG2a (0.25 mg/kg) or mIgG2a isotype (0.25 mg/kg). N = 10 per group. (*D*) Tumor-free mice that were previously treated with JTX-1011-mG2a were re-challenged s.c. on the opposite flank with Sa1/N tumors ~45 days after the final dose of antibody was administered (N = 7 for each). Naïve mice were challenged with Sa1/N as a positive control (N = 10). Kaplan-Meier tumor-free survival curves shown. *** *P<0*.*001* compared to isotype. (*E*) Tumor-growth curves of mice treated with 37A10-mG2a (0.25 mg/kg) or 37A10-mIgG1-agly (0.25 mg/kg), or mIgG2a and mIgG1 isotype (5.0 mg/kg each). N = 10 per group. In tumor growth curves, each line indicates an individual mouse, and number of tumor-free mice out of 10 at the end of study is indicated for each group.

We first focused on the Sa1/N fibrosarcoma model, because the therapeutic potential of targeting ICOS using an ICOS ligand-based therapy had been previously shown in this model [25, 26]. Tumor-bearing A/J mice were randomized by tumor size when tumors reached an average volume of 100 mm^3^ (day 7 post-inoculation), and the mice then received a total of 4 doses of antibody administered twice a week (0.25–0.3 mg/kg) beginning at the day of randomization (Fig 3A). As shown in Fig 3B, treatment with 37A10-mG2a resulted in complete tumor regression in 9/10 mice as compared to 0/10 in the isotype treated group. JTX-1011-mG2a was likewise tested in the Sa1/N model with the same dosing schedule. Similar to 37A10-mIgG2a, administration of JTX-1011-mG2a resulted in anti-tumor activity with 7 out of 10 mice showing complete tumor regression (Fig 3C).

One of the hallmarks of immunotherapy is induction of memory responses. We therefore assessed whether ICOS antibody treatment could induce long-term protection in mice. Tumor-free mice previously treated with JTX-1011-mG2a were re-challenged with Sa1/N tumors on the contralateral flank ~45 days after the last antibody treatment. While tumors developed in 100% of untreated naïve control mice, there was no tumor growth in the ICOS antibody-treated groups (Fig 3D), indicating that systemic long-term immunity was induced in these mice.

As *in vitro* studies indicated that ICOS antibodies need to be plate-bound or cross-linked for an optimal agonist signal, we assessed the role of the Fc portion of the ICOS antibody in *in vivo* tumor studies. For this purpose, we generated 37A10-mIgG1-agly, a chimera of hamster V regions with an aglycosylated mouse IgG1, which lacks the ability to bind Fcγ receptors (FcR). In contrast to 37A10-mIgG2a, administration of 37A10-mIgG1-agly had a much-reduced effect on tumor growth (Fig 3E), indicating that the Fc portion of the ICOS antibody is critical for optimal anti-tumor effect. An important role of FcR binding was previously shown for other therapeutic antibodies, including anti-CD40, anti-CTLA4 and others [25, 27–29]. In agreement with the requirement for cross-linking in our *in vitro* studies (Fig 1C and 1D), FcR binding can serve as a scaffold to cross-link ICOS antibodies leading to oligomerization of ICOS receptors and proximal signaling. In addition, the FcR binding can enable effector functions such as antibody-dependent cell-mediated cytotoxicity (ADCC) which may also explain the reduced activity of the aglycosylated antibody.

### ICOS antibodies are efficacious as monotherapy and in combination with anti-PD-1 and anti-CTLA-4 in multiple mouse syngeneic tumor models

**Fig 4:**
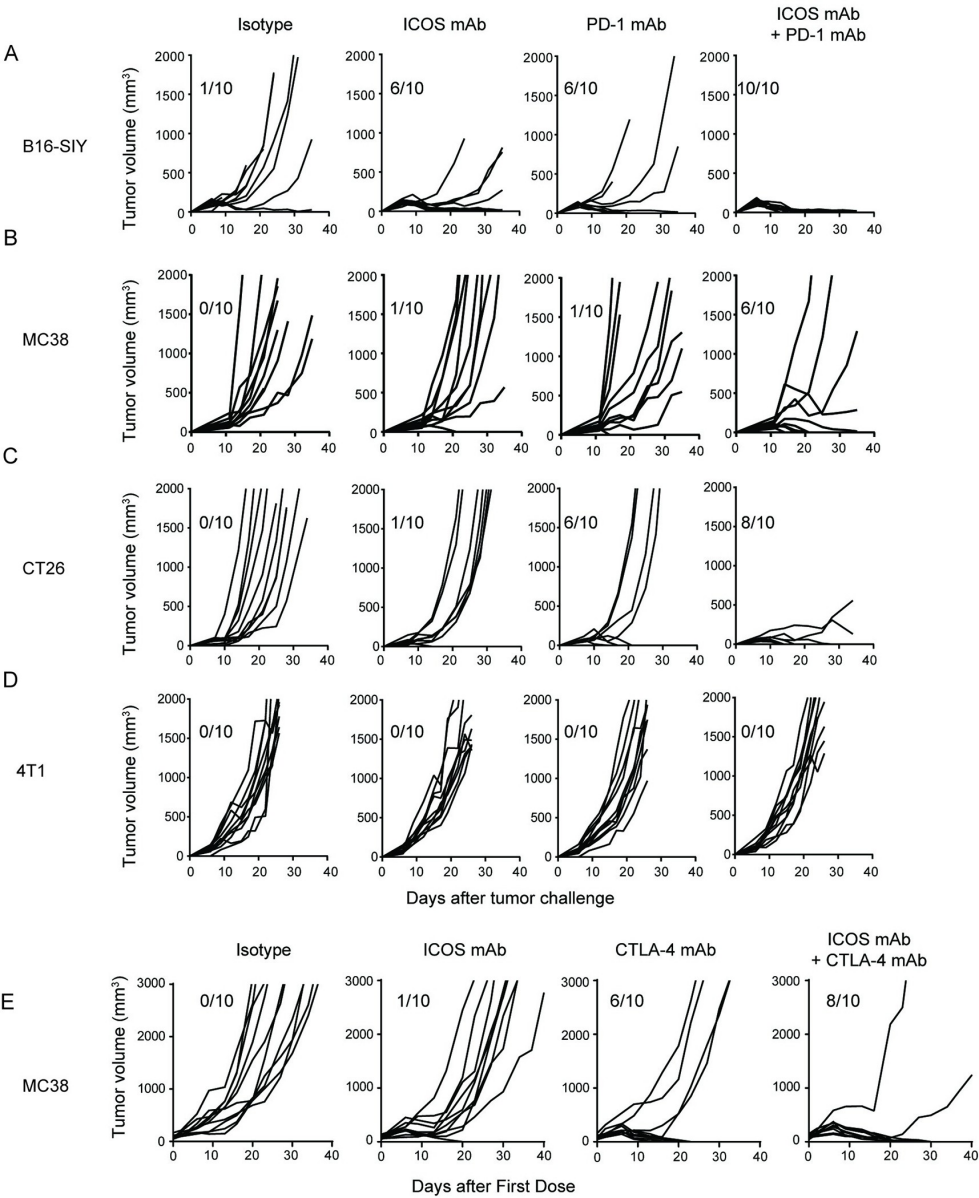
A-D: Effect of combination treatment with ICOS and anti-PD-1 antibodies in multiple syngeneic tumor models. Mice received 0.25 mg/kg of ICOS antibody (ICOS mAb) and/or 10 mg/kg of anti-PD-1 antibody RMP1-14 (PD-1 mAb) administered i.p. Treatment in CT26 started on day 3 after tumor challenge when tumors were palpable, and antibodies were administered twice weekly for two weeks. Treatment in the 4T1 model started on day 3 after tumor challenge when tumors were palpable, and antibodies were administered once weekly for two weeks. Treatment in the B16-SIY and MC38 models started on day 6 and 11, respectively, after tumor challenge when tumors reached an average volume of ~100 mm^3^, and antibodies were administered once weekly for two weeks. E. Effect of combination treatment with ICOS and anti-CTLA-4 antibodies in the MC38 syngeneic tumor model in huCTLA-4 knock-in mice. Treatment in MC38 began when tumors reached an average volume of 80mm^3^; ipilimumab was administered i.v. once at the time of randomization, and ICOS antibody was administered i.p. at day of randomization and 7 days post-randomization. Each line indicates an individual mouse. Numbers of tumor-free mice out of 10 at the end of study are indicated for each group.

We next assessed the effect of ICOS antibody treatment in several additional syngeneic tumor models, both as a monotherapy and in combination with an anti-PD-1 antibody (Fig 4). Assessment of efficacy in a range of tumor models is important given the heterogeneity of human cancers; furthermore, evaluation of the ICOS antibody in combination with a PD-1 inhibitor is highly relevant given the current clinical landscape of cancer immunotherapies. As a single agent, treatment with ICOS antibodies resulted in significant tumor regression in the B16-SIY model (Fig 4A; 6/10 tumor-free), but little/no tumor regression observed in the MC38 (Fig 4B), CT26 (Fig 4C), or 4T1 (Fig 4D) models. Likewise, the anti-PD-1 antibody only showed single agent activity in a subset of the models, namely in B16-SIY and CT-26, with little/no tumor regression observed in MC-38 or 4T1. In the B16-SIY model, while anti-PD-1 or ICOS antibody alone resulted in 6/10 tumor-free mice, combination treatment resulted in complete regression in 10/10 mice. In the MC38 model, monotherapy with anti-PD-1 or ICOS antibody provided minimal anti-tumor benefit (1/10 tumor-free); however, combination therapy resulted in tumor regression in 6/10 mice. A combination effect was also observed in the CT26 model, resulting in 8/10 tumor-free mice and inhibition of tumor growth in all of the mice in the combination group. Neither monotherapy with either antibody alone nor combination therapy resulted in any anti-tumor effect in the 4T1 model. Preclinical and clinical studies utilizing anti-CTLA4 antibodies in ICOS or ICOS-L knockout mice have demonstrated the critical role of ICOS in anti-CTLA-4 activity, so we also sought to evaluate if ICOS agonism may enhance efficacy of anti-CTLA-4 antibodies in vivo. Given the reported differences in the mechanism of action of the human anti-CTLA-4 antibody ipilimumab and mouse surrogate anti-CTLA-4 antibodies [29–31], we used a knock-in mouse strain (huCTLA-4) expressing a humanized version of CTLA-4. Relative to the significant level of Treg depletion that has been demonstrated in the literature using commercially available mouse-reactive clones, this model system has the advantage of minimizing the anti-CTLA-4-mediated Treg depletion that is commonly observed with surrogate antibodies (S3 Fig in S1 File). Treatment of huCTLA-4 mice bearing MC38 tumors with ipilimumab alone resulted in 6/10 tumor free mice, and the combination of ipilimumab and ICOS agonist increased this to 8/10 with tumor growth inhibition observed in one additional mouse (Fig 4E). These data suggest that ICOS agonist antibodies may enhance the effect of both anti-PD-1 and anti-CTLA-4 antibodies when administered in combination.

Similar to what was previously described in the Sa1/N model, we assessed the durability of observed anti-tumor responses in additional tumor models. Mice previously treated with JTX-1011-mG2a (from study in Fig 4A) who had rejected their original B16-SIY tumors were re-challenged with B16-SIY tumor cells approximately 10 weeks following their last treatment. Mice previously cured displayed no tumor growth and survived re-challenge (S4A and S4B Figs in S1 File). To assess the specificity of the immune response, we implanted B16-SIY tumor cells and treated a separate cohort of mice for profiling of peripheral blood and spleen T cell responses following isotype or JTX-1011-mG2a treatment. Treatment with JTX-1011-mG2a resulted in proliferation of T cells in both peripheral blood and spleen as assessed by Ki67 staining (S4C Fig in S1 File). To assess the antigen-specificity, commercially available B16-SIY tetramers were utilized. Flow profiling demonstrated increased proliferation of tetramer+ T cells in the spleen of JTX-1011-mG2a treated mice (S4D Fig in S1 File) as well as increase in the frequency of activated memory CD8 T cells in mice treated with JTX-1011-mG2a relative to isotype (S4E Fig in S1 File), demonstrating induction of anti-tumor immunity in the ICOS agonist treated mice. A similar re-challenge study was performed in mice who had previously rejected CT26 tumors following treatment with JTX-1011-mG2a in combination with anti-PD-1. Mice were challenged with CT26 tumor cells as well as EMT6 cells on the opposite flank. Rapid rejection of CT26 cells but outgrowth of EMT6 tumors demonstrates specificity of induced immune responses (S4F Fig in S1 File).

### Characterization of peripheral and intratumoral immune cell composition during treatment

**Fig 5:**
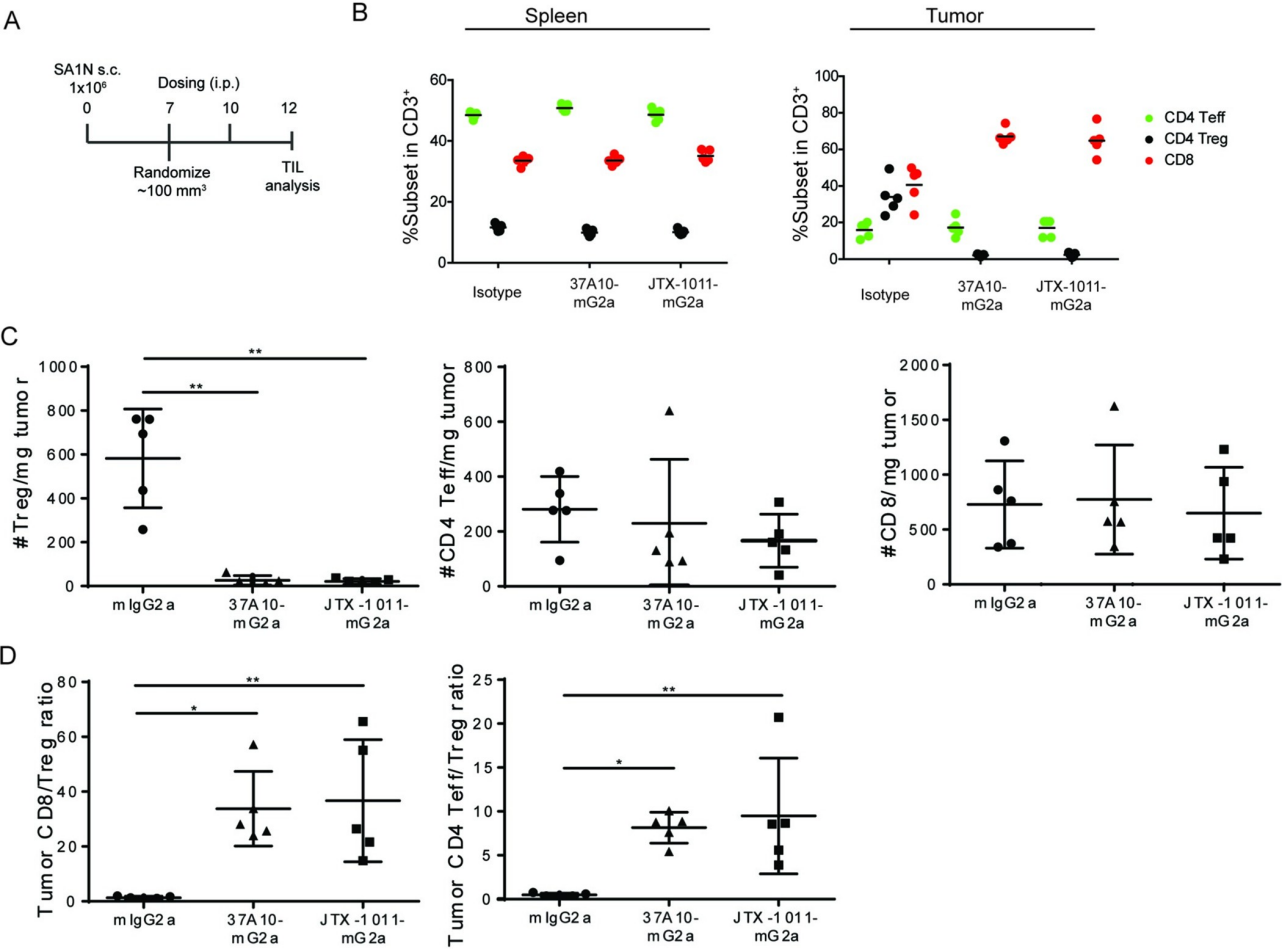
Changes in T cell subsets in tumors following ICOS antibody administration *in vivo*. (*A*) Dosing regimen for TIL studies in Sa1/N model. Dosing frequency and timing are the same as in Fig 3A. Single cell suspensions from spleens and tumors analyzed by flow cytometry 2 days after the second dose. (*B*) Frequencies of T cell subsets in CD3^+^ cells in spleens (left), frequencies of T cell subsets in CD45^+^CD3^+^ cells within tumors (right) (N = 5 per group). (*C*) Number of Tregs (left), CD4 Teff (middle) and CD8 T cells (right) per milligram (mg) of tumor. (*D*) CD8:Tregs (left) and CD4 Teff:Tregs (right) ratios in tumors after treatment. Each symbol indicates an individual mouse or donor; lines indicate mean ± SD.

To further elucidate the effect of ICOS antibody administration on immune cell composition, we next analyzed the changes in tumor infiltrating leukocytes in Sa1/N tumors following treatment. For this purpose, spleens and tumors were harvested 2 days following administration of the second dose of 37A10-mG2a, JTX-1011-mG2a or mIgG2a isotype (0.25 mg/kg), and single cell suspensions were analyzed by flow cytometry (Fig 5A and S5A Fig in S1 File). We found no differences in the frequency of T cell subsets within CD3^+^ cells in the spleens of isotype or ICOS antibody treated mice (Fig 5B and S5B Fig in S1 File). However, the percentage of intratumoral CD4^+^FoxP3^+^ Treg within CD3^+^ cells decreased from 38% in the isotype-treated group to 3.3% in the 37A10-mG2a treated group (S5B Fig in S1 File). A similar decrease was observed with JTX-1011-mG2a treatment (Fig 5B). An increase in frequency of CD8 T cells in tumors was observed upon ICOS antibody treatment; however, the frequency of CD4^+^FoxP3^-^ Teff cells was not affected (Fig 5B and S5B Fig in S1 File). To confirm whether the changes in frequencies reflected changes in the abundance of T cells subsets, we measured the absolute number of cells relative to the weight of the tumors. While there was no significant change in the number of CD8 and CD4^+^ Teff cells, ICOS antibody treatment led to a dramatic decrease in the number of Tregs (Fig 5C and S5C Fig in S1 File). These changes resulted in an overall increase in the CD8:Treg (26.5x) and CD4 Teff:Treg (7.8x) ratios in the tumors (S5D Fig in S1 File), with similar changes observed with JTX-1011-mG2a treatment (Fig 5D). Interestingly, while absolute numbers of CD4 Teff cells were unaffected, treatment with the ICOS antibody 37A10-mG2a significantly enhanced Ki-67 staining on CD4 Teff cells in the tumor but not in the spleen (S5E Fig in S1 File). In a similar study in the B16-SIY model, we detected a decrease in the frequency of Tregs in tumors but not in spleens, providing further evidence that this effect is specific to tumor microenvironment (S6A Fig in S1 File). Similar to Sa1/N tumors, JTX-1011-mG2a treatment in B16-SIY tumors resulted in Treg depletion and an increase in effector T cell to Treg ratios (S6B Fig in S1 File). We also analyzed other immune cell types including CD11b^+^Ly6C^+^ and Ly6C^-^ myeloid cells, CD11b^+^ Ly6G^+^ granulocytes, CD19^+^ B cells and CD49b^+^ NK cells in spleens and Sa1/N tumors and found little to no changes in these cell populations (S6C Fig in S1 File). Taken together, the lack of a reduction in Tregs in the periphery indicated that the activity of JTX-2011 on Tregs is tumor microenvironment-specific.

Analysis of ICOS levels on different T cell subsets *ex vivo* indicated that Tregs express the highest level of ICOS, followed by CD4 Teff, and then CD8 T cells (S5F and S5G Figs in S1 File). Notably, ICOS expression on T cell subsets in the periphery are relatively low compared to expression in mouse and human tumors (S5F and S5G Figs in S1 File). While ICOS signaling has been shown to play a role in the development and homeostasis of Tregs [32, 33], we did not observe an increase in Treg populations following agonist antibody treatment. The importance of antigen expression level for ADCC has been previously shown for other monoclonal antibodies [34, 35], and the high level of ICOS expression on intratumoral Tregs may account, in part, for the specific depletion. Moreover, the tumor-centric reduction in Tregs may be a function of the interaction of ICOS antibodies with specific FcRs that are more abundant in cells present in the tumor microenvironment. This effect is similar to what was described regarding Treg depletion after anti-CTLA-4 treatment which was shown to be mediated by FcgRIV expressed on myeloid cells in tumors [29]. This observation is likely a mouse-specific phenomenon as murine models are well regarded as exaggerating ADCC. The depletion of Treg cells by anti-CTLA-4 (ipilimumab) observed in preclinical studies did not translate to Treg depletion in humans [31]. Similarly, clinical data with JTX-2011 demonstrated no Treg depletion either in peripheral blood or intratumorally in the Phase 1/2 ICONIC study [36].

ICOS has long been recognized as a critical regulator of humoral immune responses, with ICOS and ICOS-L mediating T and B cell interactions within germinal centers contributing to the induction of humoral immunity. Originally studied in infectious diseases and autoimmunity, the observation of ICOS hi CD4 T cells emerging where durable clinical benefit was observed in melanoma subjects treated with ipilimumab, brought ICOS into focus as potentially driving durable responses in oncology.

ICOS is a member of the B7/CD28 family of costimulatory molecules, which include PD-1, CTLA-4, and CD28. Additional T cell costimulatory molecules such as OX-40, 4-1BB, CD40, and CD28 have been demonstrated to require ICOS signaling to exert their biological function, suggesting ICOS regulates a critical nexus of costimulatory activity [10, 11, 13, 14]. This report describes the generation of the ICOS antibody JTX-2011, and our preclinical studies suggested a dual mechanism of action, whereby it exerted an agonist effect on T effector cells and specific reduction of Tregs within the tumor. The Treg depletion mechanism has not translated to humans and may be a mouse-specific phenomenon, as Treg depletion has not been observed in human subjects treated with JTX-2011 in the ICONIC trial [37]. Importantly, the ICOS antibodies described herein require initial TCR priming to induce ICOS expression for activity and thereby lack superagonistic effects. Our ICOS agonist antibodies do not cross-react with other B7/CD28 members that have been associated with superagonism.

Treatment with the described ICOS agonists *in vivo* induced tumor regression in multiple tumor models and promoted long-term immunity. The anti-tumor activity of the ICOS antibody was further enhanced in combination with anti-PD-1 in multiple tumor models as well as with the anti-CTLA-4 antibody ipilimumab. Given the robust anti-tumor activity of the ICOS antibody in preclinical models together with the promising safety profile, human clinical trials of JTX-2011 are being conducted.

## Methods

### Generation of ICOS constructs and cell lines

Human ICOS-Fc was generated at Precision Antibody (Columbia, MD) by subcloning the extracellular domain of human ICOS into pINFUSE-hIgG1-Fc1 vector and transient production in Cos7 cells. Recombinant monomeric mouse and human ICOS-Fc proteins were generated by co-expression in HEK293 cells of a vector containing ICOS-Fc with a C-terminal C-tag and a vector containing Fc alone with a C-terminal His tag. Monomeric ICOS-Fc heterodimers were isolated by sequential C-tag and His6 tag purification.

CHO cells expressing mouse ICOS were generated using pCMV6 vector (Origene). For human, rat and cynomolgus ICOS expressing cells, full-length ICOS genes were cloned into pcDNA3.1 vector. For the Jurkat reporter assay, chimeric constructs comprised of the extracellular and transmembrane domains of either human ICOS (NCBI NM_012092.3, amino acids 1–161) or mouse ICOS (NCBI NM_017480.2, amino acids 1–165) fused with the cytoplasmic domain of human CD28 (NCBI_006139.3, Uniprot P10747, amino acids 180–220) were synthesized at DNA2.0, and subcloned into multiple cloning site of pCDH-EF1-MCS-IRES-PURO lentiviral vector (SBI Bioscience). Lentivirus expressing the ICOS constructs were transduced into Jurkat NFκB-GFP reporter cells (SBI Bioscience) to generate the ICOS-expressing reporter cell lines.

### Generation of ICOS antibodies

ICOS monoclonal antibodies were selected from a hybridoma library generated at Precision Antibody following immunization of Armenian hamsters with human ICOS-Fc. Hybridomas were screened for binding to CHO-hICOS or CHO-mICOS cells, and for activity in a Jurkat NF-κB-GFP reporter assay. Clones of interest were expanded and then purified by Protein A chromatography (GE Healthcare). To generate recombinant versions of 37A10, the variable regions of antibody were sequenced from the hybridomas to determine antibody identity (LakePharma; Belmont, CA). Chimeric versions of 37A10 comprising the hamster variable regions and murine IgG1 and murine IgG2a constant regions were generated by subcloning the variable regions into pTT5 expression vectors (National Research Council Canada) containing the mouse heavy chain and the mouse kappa light chain. An aglycosylated version of the murine IgG1 constant region (mIgG1 agly) contains a N297A point mutation to abrogate FcγR binding. 37A10 mIgG chimeras were generated by transient transfection of HEK293 cells and purified by Protein A affinity chromatography. Humanized variants of 37A10 were generated by selecting human germline acceptor frameworks with high identity to the 37A10 hamster parent and identifying a small number of positions within the parental hamster framework predicted to be potentially important for antigen binding or stability. Variant heavy chains and light chains were expressed combinatorially and screened for retention of binding to recombinant monovalent ICOS-Fc by ForteBio Octet and to CHO-ICOS cells by flow cytometry as described below.

The binding affinities of ICOS antibodies to recombinant monomeric mouse and human ICOS-Fc were determined using ForteBio Octet. Briefly, ICOS antibodies were loaded onto the appropriate biosensors (ForteBio) at 10 μg/mL. Loaded biosensors were then dipped into wells containing the monomeric ICOS-Fc at concentrations up to 30.0 μg/mL for the association step, and subsequently, into kinetics buffer (PBS with 0.1% BSA, 0.05% sodium azide, 0.02% Tween-20) for the dissociation step. Binding constants were derived from a global 1:1 fitting model using the ForteBio Data Analysis software (v8.2). Affinity of JTX-2011 and other biologics to CD28 family receptors was tested in a similar way using ForteBio Octet. For this purpose, anti-hBTLA clone 330104 (Abcam), anti-mBTLA clone 6F2 (eBioscience), hPD-1-Fc, mPD-1-Fc, anti-hPD-1 (Jounce Therapeutics), hB7-1-Fc, hBTLA-Fc hCD28-Fc, hCTLA4-Fc, mB7-H1-Fc, mBTLA-Fc, mCD28-Fc and mCTLA4-Fc (R&D Systems) were used. For cell binding, CHO-ICOS cells (5x10^4^/well) were incubated with 50 μl of ICOS antibodies at indicated concentrations in PBS at 4˚C for 30 min in duplicates. Cells were washed twice with PBS and stained with flurochrome-conjugated species-specific anti-IgG antibody for 30 min at 4˚C. Cells were washed and analyzed using a BD Fortessa flow cytometer and FlowJo software.

### Jurkat NF-κB-GFP ICOS reporter assays

Jurkat-hICOS reporter cells were used at 5x10^4^/well in 96-well round bottom plates, in duplicate for each condition. The assay was performed in RMPI supplemented with 10% fetal bovine serum. PMA (Sigma-Aldrich) was used at a final concentration of 0.25ng/mL in all conditions except in unstimulated controls. Positive control anti-CD28 antibody (BioLegend) was added in soluble form at a final concentration of 66.7nM. ICOS antibodies and negative control anti-RSV-hG1 were tested in soluble form starting at 166.67nM and serially diluted 3-fold up to 11 points. Cells were cultured at 37ºC for 5hr, washed with FACS buffer (PBS supplemented with 1% fetal bovine serum, 0.01% sodium azide and 2mM EDTA) and resuspended in 100μL of FACS buffer. Cell were analyzed by flow cytometer.

### Primary T cell activation assays

Whole blood collected from healthy volunteers in Na-Heparin vacutainer tubes was obtained from Research Blood Components and was aseptically diluted 1:2 with sterile PBS and layered over an equal volume of Ficoll-paque Plus density separation media (GE Healthcare) in 50mL conical tubes. Tubes were spun at 1000g for 30min in a bench-top centrifuge and allowed to stop without braking. The mononuclear cell layer at the plasma-Ficoll interface was collected using a sterile transfer pipette and placed into a sterile 50mL conical tube. Lymphocytes were washed three times with excess sterile PBS. Following washing, cells were counted and banked at -80ºC freezers in FBS with 10% dimethyl sulfoxide (Sigma Aldrich).

For CD4 Proliferation assays, CD4 T cells were isolated from frozen PBMC stocks using EasySep negative selection kits (StemCell Technologies). CD4 T cells were incubated with 1mL of 2.5μM CFSE dye (Life Technologies) per 2.5x107 cells in PBS for 10min at 37ºC. Reaction was stopped by addition of equal volume of FBS. Cells were washed and resuspended in culture media (RMPI supplemented with 10% fetal bovine serum). Ninety-six well U-bottom plates were coated with anti-CD3 OKT3 (6.67nM, BioLegend) alone or together with anti-RSV-hG1 Isotype control (133.4nM, Jounce Therapeutics produced based on the palivizumab sequence) or ICOS antibody (starting at 133.4nM and serially diluted 2-fold up to 10 points) in a total of 100μL PBS per well for 2hr at 37ºC. Wells were washed twice with PBS. CFSE-labeled CD4 T cells were added to wells at 1x10^5^/well. Soluble antibodies including anti-CD28 clone CD28.2 (BioLegend), anti-RSV-hG1, and JTX-2011 were added at a final concentration of 66.7nM in culture media. All conditions were tested in duplicates in a total volume of 200μL/well. Cells were incubated at 37ºC for 3 days. Cells were spun at 400xg for 5min and supernatant was saved for analysis of IFNγ levels using Cytokine Bead Arrays (BD Biosciences) based on the manufacturer’s protocol. Cells were washed and stained with fluorochrome-conjugated anti-CD4 antibody (OKT-4, 1:200, BioLegend) and dead cell discriminating Viability dye (1:1000, eBioscience) in FACS buffer for 30min at 4ºC. After washing twice, cells were resuspended in FACS buffer containing 1.5% paraformaldehyde (Alfa-Aesar) and analyzed by flow cytometry. Percentage of divided cells were determined by CFSE dilution in live CD4-gated cells.

Three versions of the cytokine release assays were performed as described below.

In the PBMC solid-phase format, JTX-2011, Herceptin®, anti-CD28.1 and anti-CD3 were prepared as a 1x solution in PBS, serially diluted 10-fold for a 5-point curve, and 100μL of each dilution was added to designated wells of a 96-well, clear, round-bottom tissue culture-treated plate. Plates were incubated overnight at 4ºC to allow proteins to bind to the plate. After the incubation period, plates were washed 3x with 200μL PBS to remove unbound protein. PBMCs were counted and adjusted to a concentration of 2x10^6^ cells/mL, and 1x10^5^ cells/50μL was added to all wells of the coated plate. 50μL of assay media (RPMI + Glutamax, 10% fetal bovine serum) was then added to all wells so that the final volume per well was 100μL.

Using the PBMC soluble-phase form, PBMCs were counted and adjusted to a concentration of 2x10^6^ cells/mL, and 1x10^5^ cells/50μL were added to all wells of a 96-well, clear, round-bottom, tissue culture-treated plate. All antibodies were prepared as a 2x concentrated solution in assay media and serially diluted 10-fold for a 5-point curve. 50μL of each dilution was added to designated wells of the plate. The final well volume was 100μL.

Fresh heparinized human whole blood was purchased commercially from Research Blood Components. In the whole blood soluble-phase form, 200μL fresh heparinized whole blood was added to all wells of a 96-well, clear, round-bottom, tissue culture-treated plate. All antibodies were prepared as a 5x concentrated solution in RPMI media and serially diluted 10-fold for a 5-point curve. 50μL of each dilution was added to designated wells of the plate. The final well volume was 250μL.

All plates were incubated at 37ºC for 24hr. After the incubation, plates were centrifuged at 400xg for 5min to pellet cells, and the supernatants were collected and frozen. Supernatants were then assayed for cytokine content on the MSD Discovery platform using the V-PLEX proinflammatory panel 1 (human) kit according to the manufacturer’s instructions.

For analysis of phosphorylated protein levels, twenty-four well plates were coated with anti-CD3 clone OKT3 (6.67nM) in a total of 500μL PBS per well for 2hr at 37ºC. Wells were washed twice with PBS. Unlabeled CD4 T cells were added to wells at 2x10^6^/mL in culture media (RPMI supplemented with 10% fetal bovine serum) in the presence of soluble anti-CD28 clone CD28.2 (66.7nM). After 24hr incubation at 37ºC, cells were washed twice with culture media. Cells were resuspended in fresh culture media and rested for 24hr at 37ºC without any stimulation. Cells were counted and added to 96-well U-bottom plates at 5x10^4^/well and incubated with 100μL/well ICOS antibody or negative control Herceptin® (66.7nM) in PBS containing dead cell discriminating Viability dye (1:1000) for 15min on ice. After washing twice with PBS, cells were resuspended in 200μL/well of complete media or complete media containing 133.4nM goat anti-human-IgG secondary antibody (“cross-linker”, Jackson Immunoresearch). After incubation at 37ºC for 2, 5 or 30min, 50μL of 5X Lyse/Fix Buffer (BD Biosciences) was added in each well, and cells were incubated for another 10min at 37ºC. Cells were washed, resuspended in 100μL of ice-cold Perm Buffer III (BD Biosciences), and incubated on ice for 30min. After two washes with FACS buffer, cells were stained with fluorochrome-conjugated anti-pAKT S473/D9E, anti-pERK1/2 T202/Y204 or isotype control antibodies (Cell Signaling Technologies) in FACS buffer for 45min at room temperature in dark. Cells were washed twice and resuspended in FACS buffer containing 1.5% paraformaldehyde and analyzed by flow cytometry. Percentage of pAKT positive cells were determined in live-gated cells.

### Mice

Female A/J, Balb/c and C57BL/6 mice were obtained from Jackson Laboratories at 6–8 weeks of age. Human CTLA-4 knock-in (huCTLA-4) mice were generated at Crown Biosciences (Beijing, China) by replacing mouse CTLA-4 exons 2 and 3 with their human counterparts. For all animal studies conducted in house, mice were maintained in accordance with the Jounce Therapeutics Institutional Animal Care and Use Committee (IACUC) protocols e Mice were housed 2–5 per cage in specific pathogen free conditions, given water and chow ad libitum and were allowed to acclimate 5–7 days after arrival before being enrolled in a study. Animal handling was performed by trained personnel using all efforts to minimize suffering and distress. In house tumor studies conducted according to Jounce animal protocol JT02-13 and were approved by the Jounce IACUC. Subcutaneous tumor implantation was performed under isoflurane anesthesia (2% isoflurane in O_2_). Tumor growth and animal body weights were monitored at least twice weekly. Mice were sacrificed when tumor volumes exceeded 2000mm^3^, tumor became ulcerated, body weight decreased by at least 20% or other signs of clinical distress were noted, in accordance with Jounce protocol JT02-13. Euthanasia was performed by CO2 inhalation followed by cervical dislocation as secondary means.

MC38 syngeneic studies with huCTLA-4 knock-in mice were conducted by Crown Biosciences and were approved by their institutional IACUC. During the study, the care and use of animals was conducted in accordance with the regulations of the Association for Assessment and Accreditation of Laboratory Animal Care (AAALAC). Animals were checked daily for morbidity and mortality, tumor volume, and body weight were measured twice weekly. Mice were sacrificed when tumor volume exceeded 3000mm^3^, tumor ulcerations reached 25% of the tumor surface, body weight loss reached 20% or other signs of clinical distress were noted.

### Mouse syngeneic tumor models

4T1 breast carcinoma and CT26 colon carcinoma cell lines were obtained from ATCC. Sa1/N fibrosarcoma and MC38 colon adenocarcinoma cells were obtained from Dr. J. Allison (MD Anderson Cancer Center), and B16-SIY melanoma cells were obtained from Dr. T. Gajewski (University of Chicago). Sa1/N and B16-SIY cells were cultured in DMEM with glutamine, 1X beta-mercaptoethanol and 10% FBS. MC38, CT26 and 4T1 cells were cultured in RPMI with glutamine and 10% FBS. Cells were tested and confirmed negative for *Mycoplasma* and viral pathogens.

For tumor cell inoculation, one vial of Sa1/N cells was thawed, washed and cultured in T150 flasks in Sa1/N culture media. Cells were expanded in 2 passages. Cells were prepared by Trypsin-EDTA (Gibco) treatment for 5min at 37˚C and washed. Cells were counted, and viability was determined by trypan blue (used if viability was > = 95%). Sa1/N cells were resuspended in PBS at 1x10^7^/mL and kept on ice. 6–8 week old female A/J mice were inoculated s.c. on the right flank with 1x10^6^ Sa1/N cells in 100uL PBS using tuberculin syringes with 27-gauge needles. Tumor growth was monitored and on day 7, animals were redistributed into new cages after normalizing the average tumor volume to 100mm^3^ for each treatment group. 10 mice were included in each treatment group. Mice were dosed i.p. on days 7, 10, 14 and 17 with 5.0, 0.3, or 0.25 mg/kg of 37A10, 37A10-mG2a, JTX-1011-mG2a, 37A10-mG1-agly, or mouse IgG2a (clone C1.18.4, BioXcell) as indicated. Tumor infiltrating cells were analyzed on day 12 as described below. Tumor re-challenge studies were performed by re-inoculating mice on the left flank ~45 days after the last treatment.

One vial of MC38 cells was thawed, washed and cultured in T150 flasks in culture media. Cells were expanded in 2 passages. Cells were prepared by Trypsin-EDTA treatment for 5min at 37˚C and washed. Cells were counted, and viability was determined by trypan blue (used if viability was > = 95%). MC38 cells were resuspended in PBS at 5x10^6^/mL and kept on ice. 6–8 week old female C57Bl/6 mice were inoculated s.c. on the right flank with 5x10^5^ MC38 cells in 100uL of PBS using tuberculin syringes with 27-gauge needles. On day 6, animals were randomized by tumor volume prior to assignment to a treatment group. 10 mice were included in each treatment group with an average starting volume of 100mm^3^. Animals were administered antibodies via i.p. injections, and dosed on days 6, 9, 13 and 16.

One vial of CT26 or 4T1 cells was thawed, washed and cultured in T150 flasks in culture media. Cells were expanded in 2 passages. Cells were prepared by Trypsin-EDTA treatment for 5min at 37˚C and washed. Cells were counted, and viability was determined by trypan blue (used if viability was > = 95%). CT26 or 4T1 cells were resuspended in PBS at 1x10^6^/mL and kept on ice. 6–8 week old female Balb/c mice were inoculated s.c. on the right flank with 1x10^5^ CT26 or 4T1 cells in 100uL of PBS using tuberculin syringes with 27-gauge needles. On day 3, animals were redistributed into new cages for randomization. 10 mice were included in each treatment group. Animals were administered antibodies via i.p. injections on days 3, 6, 10 and 13 for CT26, and on days 3 and 10 for 4T1.

For in house MC38, B16-SIY, CT26 and 4T1 models, animals were treated with 0.25 mg/kg ICOS antibody (37A10-mG2a in CT26, JTX-1011-mG2a in others) and/or 10 mg/kg anti-PD-1 (RPM1-14, BioXcell) or 0.25 mg/kg mouse IgG2a plus 10 mg/kg rat IgG2a (2A3, BioXcell) isotype controls. For the study in huCTLA-4 knock-in mice, animals were treated with a single 10 mg/kg dose of either clinical grade ipilimumab (Yervoy^®^, Bristol-Meyers Squibb) or human IgG1 isotype control administered intravenously, and two weekly 0.25 mg/kg doses of ICOS antibody or mouse IgG2a isotype control administered intraperitoneally. Tumor volumes were measured using calipers and calculated using the formula [0.5 x (length x width^2^).

### Ex vivo analysis of murine and human tumors and quantification of ICOS expression

SA1/N and B16-SIY tumor analyses were performed 2 days following the second dose of antibody treatment. Tumors were weighed on a microscale on plastic weigh boats and dissociated with a 0.25mg/mL Liberase TL (Sigma-Aldrich), 0.05mg/mL DNaseI (Roche) from bovine pancreas, and RPMI Medium mixture for 20 min at 37<C. Crude tumor homogenate was mashed through a 70um cell strainer. Filters were washed, and tumor homogenate was centrifuged at 400xg for 5min. Tumor homogenate was resuspended in 250ul and distributed to round bottom 96-well plates for staining. Spleens were collected in complete RPMI (supplemented with 10% fetal bovine serum). Single cell suspensions of spleens were prepared mechanically by gently pushing cells on 70 μm filters using syringe plungers. Spleen homogenate was centrifuged at 400xg for 5min and resuspended in 1mL of ACK lysis buffer (Gibco) for 3min on ice. Five mL of media was added and RBC lysed spleen homogenate was centrifuged at 400xg for 5min and resuspended in 1mL of complete RPMI. Spleen homogenate was then distributed to round bottom 96-well plate for staining. Cells were centrifuged at 400xg for 5min and resuspended in 95ul of FACS buffer (PBS supplemented with 1% fetal bovine serum, 0.01% sodium azide and 2mM EDTA) and 5uL Mouse TruStain FcX (BioLegend) for 15min at 4ºC. Cells were centrifuged at 400xg for 5min and resuspended in 100uL of staining cocktail. Staining cocktail contained Mouse TruStain FcX (1:20), fluorescently labeled anti-CD45 (30-F11), anti-CD3 (145-2C11), anti-CD8 (53–6.7), anti-CD4 (GK1.5), anti-49b (DX5), anti-Ly6G (1A8), anti-Ly6C (AL-21), anti-Ki-67 (16A8), anti-CD11b (M1/70) and anti-CD19 (6D5) obtained from BD Biosciences or Biolegend, and Fixable Viability dye obtained from eBioscience. Staining cocktail was diluted in FACS buffer. Cells were stained for 30mins at 4ºC. Following staining, cells were washed twice with FACS buffer and resuspended in 100uL of FACS buffer supplemented with 0.1% paraformaldehyde.

To determine ICOS receptor quantity on primary cells, tumors were processed as described above. Peripheral blood was collected by cardiac puncture with 25-gauge needles and tuberculin syringes and dispersed into EDTA coated blood collection tubes. RBCs were lysed in conical tubes with 1mL of ACK lysis buffer for 3min on ice. Blood was centrifuged at 400xg for 5mins and resuspended in 100uL FACS buffer. Tumors and peripheral blood were transferred to round bottom 96-well plates and Fc blocked as described above. Following Fc block, cells were stained with 1ug/mL DyLight-650 (Life Technologies) labelled ICOS antibody 37A10 or isotype control, in addition to the staining cocktail described above. Cells were stained in 100uL of cocktail for 30mins at 4ºC. Cells were then washed twice with FACS buffer and resuspended 100uL in Fix/Perm buffer (Foxp3/Transcription Factor Staining Kit, eBioscience) and kept at 4ºC for 15mins. Cells were washed twice in Perm buffer and resuspended in 100uL of intracellular staining cocktail containing fluorescent labelled anti-FoxP3 (FJK-16s, 1:100, eBioscience) diluted in Perm buffer. Following a 30min incubation at 4ºC, cells were washed twice in FACS buffer and resuspended in 100uL of FACS buffer supplemented with 0.1% paraformaldehyde. Receptor quantitation was performed using Quantum Simply Cellular beads (Bangs Laboratories) according to manufacturer’s protocol. To determine the number of cells per milligram of tumor, a fixed number of CountBrite beads (Life Technologies) were added to samples and analyzed by flow cytometry.

For TIL analysis of MC38 tumors, huCTLA-4 mice were randomized in groups when tumor reached an average size of 153mm^3^ and treated with a single dose of clinical grade ipilimumab (Yervoy®, Bristol-Meyers Squibb) or human IgG1 isotype control administered intravenously at 10 mg/kg, or a single dose of ICOS antibody or mouse IgG2a isotype control administered intraperitoneally at 0.25 mg.kg. Mice were sacrificed 72 hours later and tumors excised, dissociated, and processed for flow cytometry analysis. The following antibodies were used: CD45-BB515 (clone 30-F11, BD), CD3-BUV395 (clone 145-2C11, BD), CD4-APCeF780 (clone GK1.5, eBiosciences), CD8-PEeF610 (clone 53–6.7, eBiosciences), FoxP3-PE (clone NRRF-30, eBiosciences), CD25-PerCP/Cy5.5 (clone PC61, Biolegend), CD19-BV605 (clone 6D5, Biolegend), CD49b-APC (clone DX5, Biolegend), ICOS-BV421 (clone C398.4A, Biolegend). Viability was assessed by staining with Fixable Viability Dye eF506 (Biolegend).

### Statistics

Parametric statistics were calculated using Student’s T Test. Non-parametric analysis was performed using Mann-Whitney U test. Multiple comparisons were calculated by ANOVA analysis. Kaplan-Meier method was used for survival analysis. EC50 values were calculated using 4-parameter logistic non-linear regression analysis. All statistical analyses were performed on GraphPad Prism software. Statistical significance indicated with * for *P<0*.*05*, ** for *P<0*.*01*, *** for *P<0*.*001* or N.S. for Not Significant.

## Supporting information

S1 File (PDF)
