# Supplementary material for: ICOS agonism by JTX-2011 (vopratelimab) requires initial T cell priming and Fc cross-linking for optimal T cell activation and anti-tumor immunity in preclinical models
Source: PLoS One. 2020 Sep 24;15(9):e0239595. doi: 10.1371/journal.pone.0239595 (PMC7514066; doi:10.1371/journal.pone.0239595)
Supplement: S1 File — (PDF) [file pone.0239595.s001.pdf]

SUPPLEMENTARY FIGURE 1

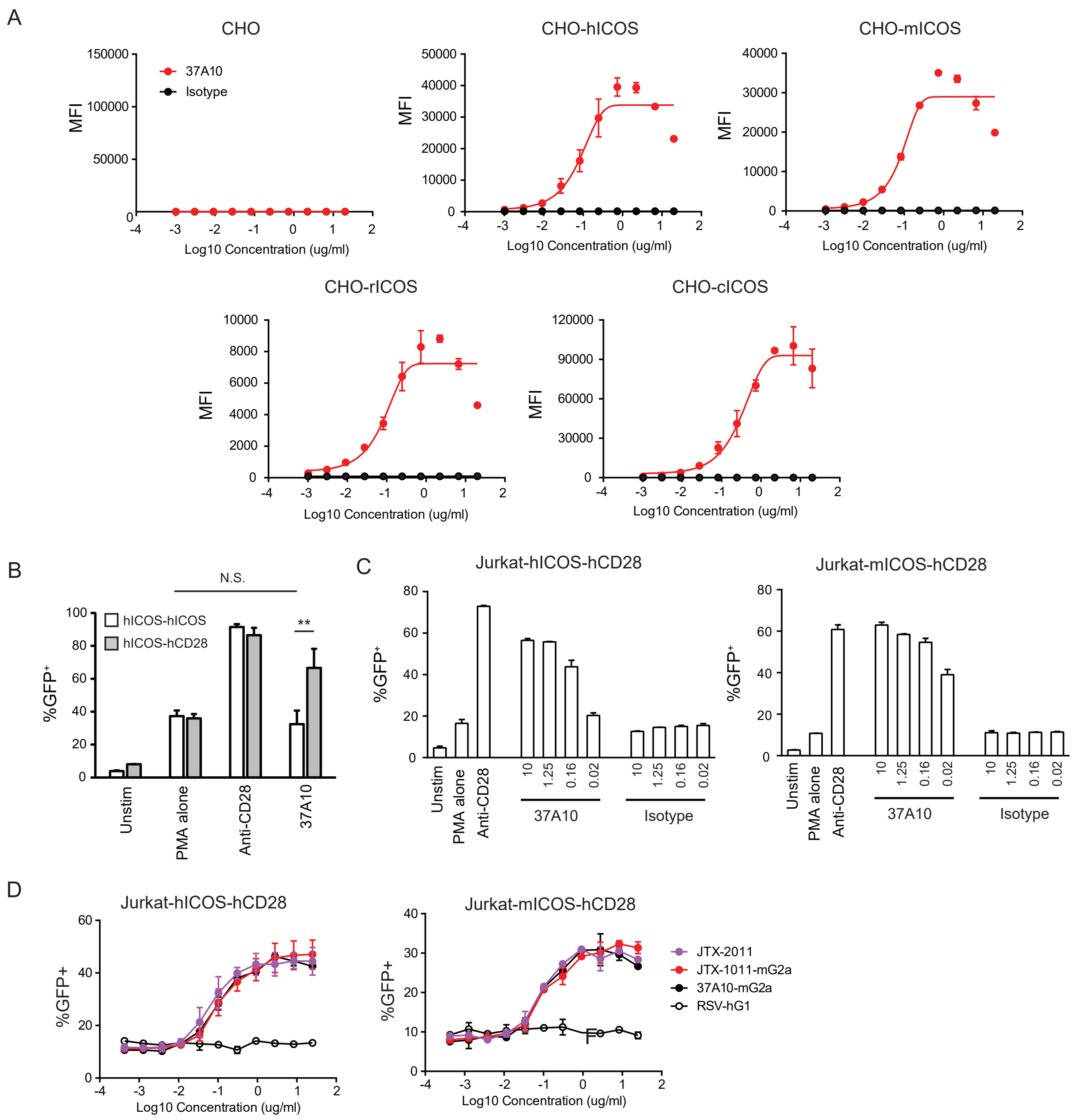

Supplementary Figure 2

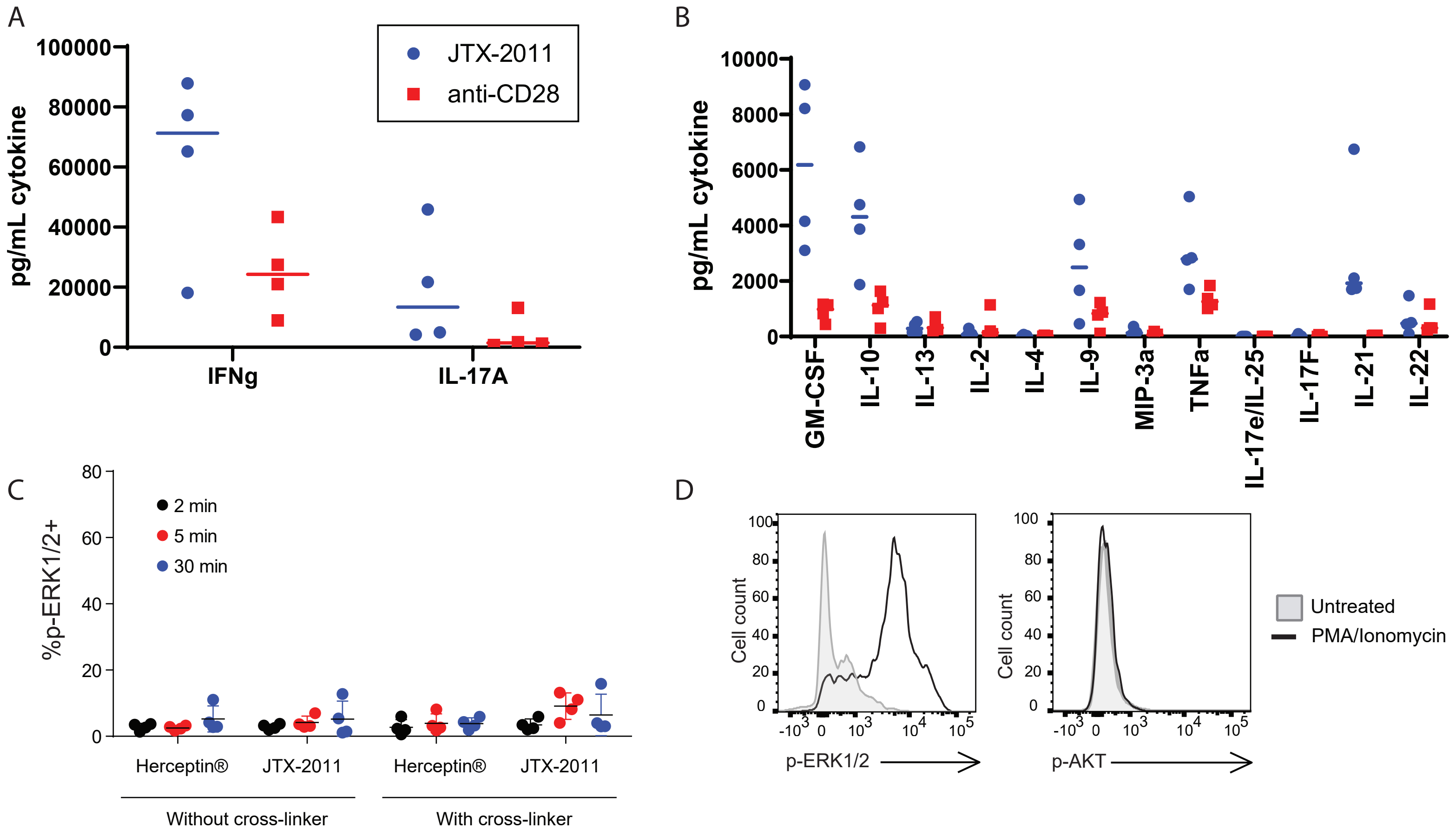

Supplementary Figure 3

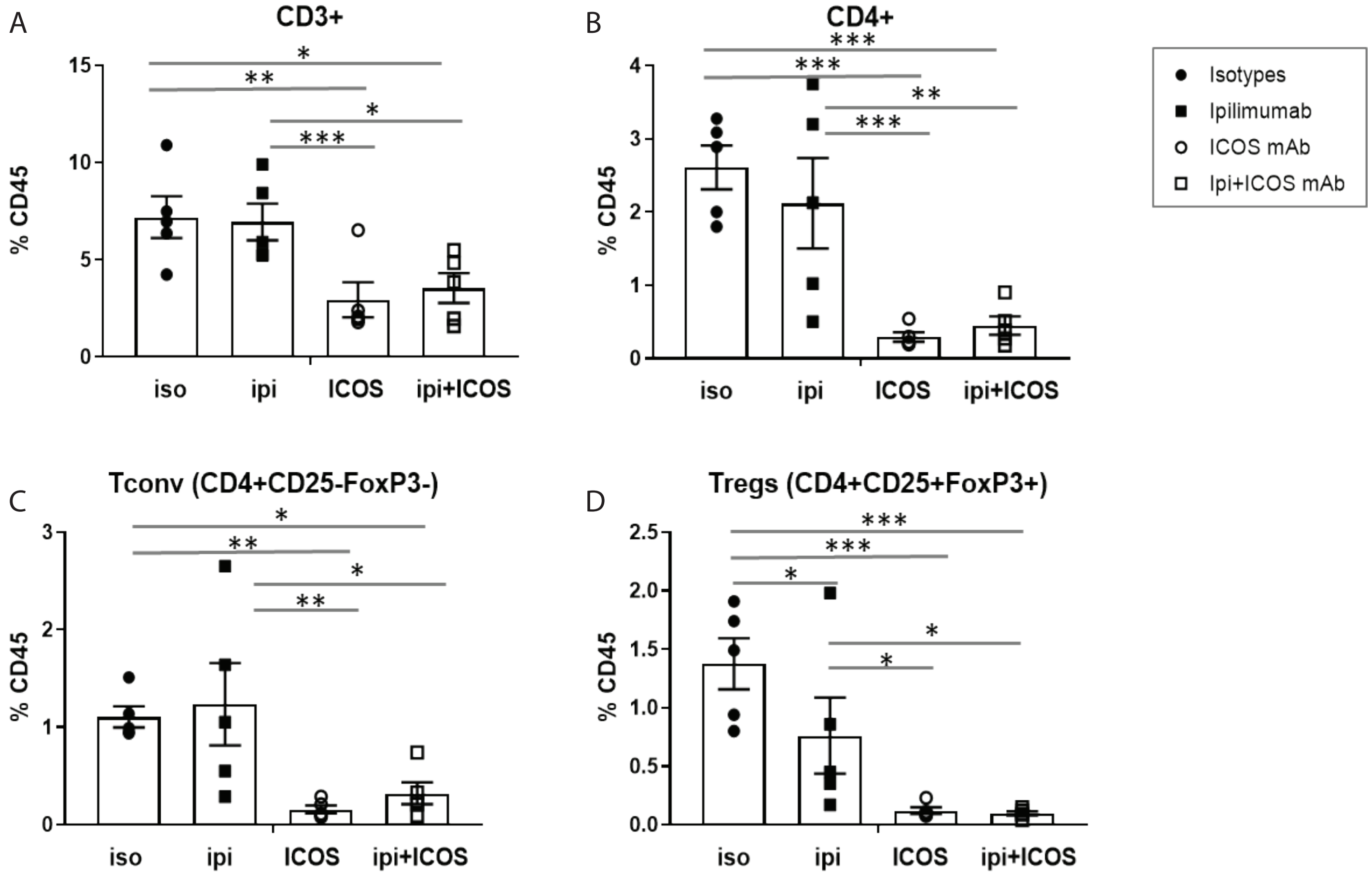

Supplementary Figure 4

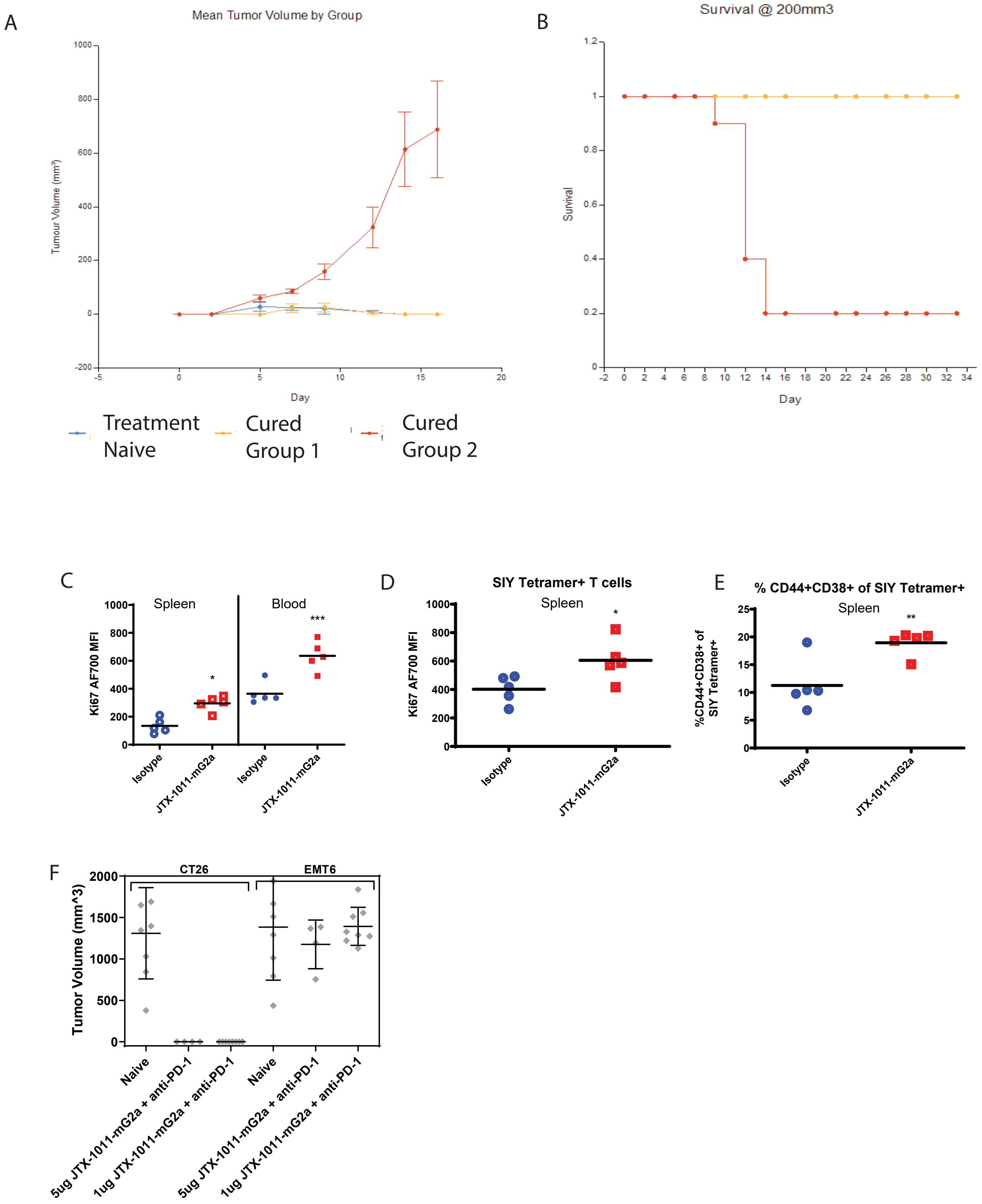

Supplementary Figure 5

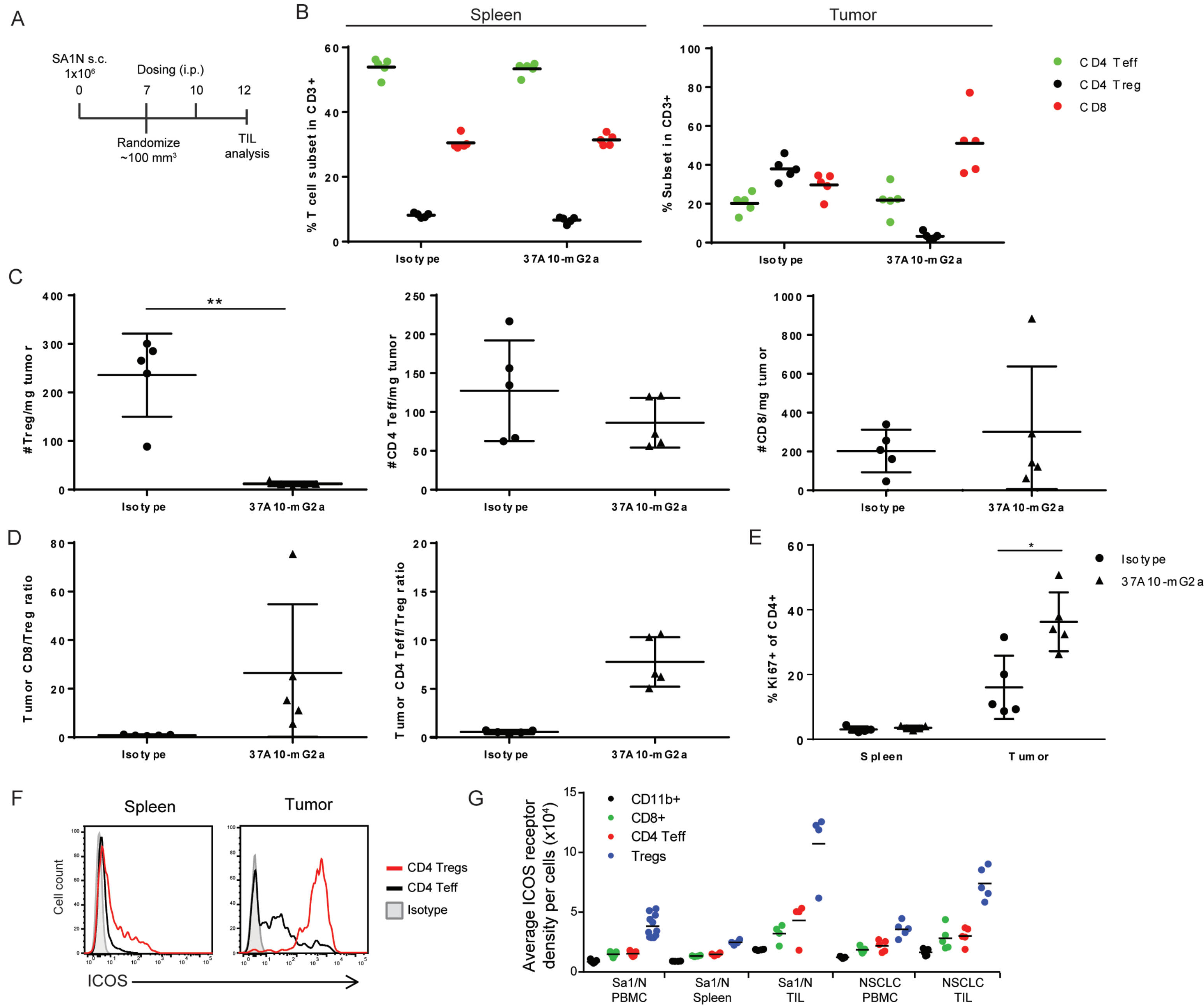

Supplementary Figure 6

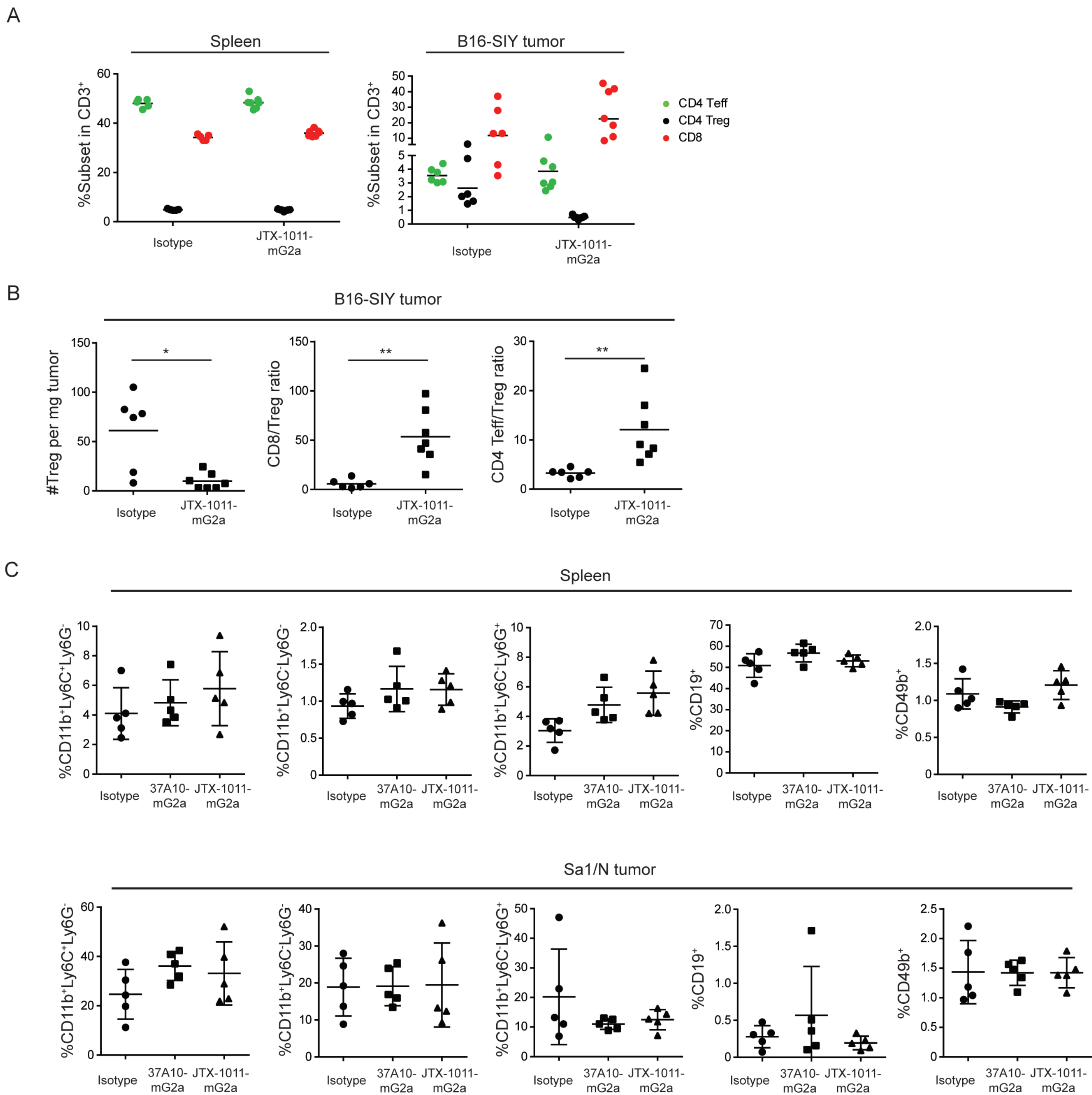

Table S1. Specificity analysis of JTX-2011 to CD28 family members.

	Affinity	
Receptor	JTX-2011	Controls[a]
hCD28-Fc	n.b.	23.0 nM
mCD28-Fc	n.b.	14.0 nM
hCTLA-4-Fc	n.b.	2.1 nM
mCTLA-4-Fc	n.b.	0.9 nM
hBTLA-Fc	n.b.	3.3 nM
mBTLA-Fc	n.b.	<1.0 nM
hPD-1-Fc	n.b.	0.5 nM
mPD-1-Fc	n.b.	36.0 nM

Binding affinity as measured by ForteBio Octet. [a] Controls are rhB7-1 for hCD28-Fc, mCD28-Fc, hCTLA4-Fc, mCTLA4-Fc; anti-hBTLA for hBTLA-Fc; anti-mBTLA for mBTLA-Fc; Nivolumab (anti-hPD-1) for hPD-1-Fc; mPD-L1 for mPD1-Fc. n.b. indicates no binding.

Table S2. Cytokine release assay. Cytokine levels in supernatants after 24 hr culture of PBMCs or whole blood with antibodies. Numbers indicate cytokine concentrations (pg/ml)

	Cytokine	Antibody concentration (nM)	CD28.1		OKT3		Herceptin®		JTX-2011	
			Mean	SD	Mean	SD	Mean	SD	Mean	SD
PBMC soluble phase	IFN-γ	666.7	4631.912	9340.58	3606.261	3487.829	6.159	10.479	9.903	11.751
		6.67	65.719	50.528	6642.145	7166.853	17.119	33.338	4.032	2.405
		0.067	28.823	57.585	7560.216	7071.766	10.209	9.174	7.07	6.742
	IL-10	666.7	43.858	34.624	94.289	60.315	1.265	2.438	0.474	0.375
		6.67	24.668	34.219	140.012	107.723	2.192	3.775	0.516	0.218
		0.067	0.827	0.361	132.455	120.859	1.087	1.449	0.54	0.3
	IL-12p70	666.7	4.49	3.72	1.706	1.342	0.118	0.218	0.114	0.154
		6.67	0.074	0.065	2.085	1.689	0.019	0.027	0.138	0.271
		0.067	0.07	0.06	2.72	1.631	0.169	0.129	0.099	0.097
	IL-13	666.7	29.746	20.625	19.372	23.105	1.094	1.33	2.114	2.326
		6.67	9.79	11.466	16.721	4.653	1.736	1.083	1.584	1.523
		0.067	1.949	1.339	16.601	8.61	2.376	2.305	1.655	1.55
	IL-1β	666.7	18.991	13.002	17.574	9.691	0.828	1.151	1.653	2.674
		6.67	1.844	2.241	20.027	13.62	0.946	1.146	0.933	1.695
		0.067	1.106	1.53	22.029	17.65	2.489	3.358	2.146	1.614
	IL-2	666.7	97.46	83.034	86.36	144.015	4.243	7.159	3.622	2.187
		6.67	29.022	28.102	65.317	27.966	3.818	5.417	2.886	1.929
		0.067	5.91	3.008	54.227	40.162	3.48	2.127	5.277	3.486
	IL-4	666.7	2.515	2.755	3.059	5.503	0.07	0.087	0.03	0.034
		6.67	1.012	2.164	1.708	0.716	0.056	0.09	0.024	0.016
		0.067	0.034	0.023	1.414	1.069	0.052	0.032	0.035	0.014
	IL-6	666.7	204.268	191.425	138.053	82.196	4.948	4.275	12.585	18.632
		6.67	18.332	12.583	84.544	49.255	4.407	2.762	6.279	6.034
		0.067	5.116	3.149	88.83	74.207	8.262	11.385	6.992	4.929
	IL-8	666.7	4474.25	2366.861	4070.978	474.237	250.823	237.136	592.36	993.997
		6.67	649.516	726.347	3438.519	1689.108	199.971	190.284	336.526	460.509
		0.067	237.646	251.79	3399.422	2130.562	562.446	936.908	554.046	674.613
	TNF-α	666.7	345.593	397.37	334.84	226.588	3.314	3.899	4.824	3.572
		6.67	19.71	18.659	314.089	211.078	3.442	3.613	3.689	2.645
		0.067	3.597	1.761	419.885	344.909	7.875	11.782	6.928	7.607
PBMC solid phase	IFN-γ	666.7	6361.332	9864.542	10434.18	9391.707	49.877	87.056	106.961	178.579
		6.67	22.369	14.439	11016.53	11538.45	1.82	1.33	4.979	5.501
		0.067	12.788	14.434	11627.3	12176.92	6.066	7.069	7.922	5.475
	IL-10	666.7	46.434	28.538	53.781	52.45	2.244	1.65	3.761	3.631
		6.67	4.179	5.611	136.615	130.928	0.641	0.299	1.01	0.548
		0.067	0.741	0.371	134.002	116.646	0.534	0.307	0.532	0.118
	IL-12p70	666.7	7.4	4.695	8.397	3.518	0.187	0.199	1.105	1.454
		6.67	0.033	0.042	4.369	3.415	0.062	0.06	0.035	0.055
		0.067	0.245	0.417	2.607	1.678	0.211	0.2	0.047	0.058
	IL-13	666.7	64.913	62.75	35.277	27.048	4.194	4.41	13.698	18.476
		6.67	4.291	2.522	22.899	19.41	1.015	1.303	2.541	2.67
		0.067	1.803	1.044	19.246	17.77	0.467	0.384	1.699	2.445
	IL-1β	666.7	83.31	135.397	44.292	30.859	3.924	3.167	9.658	12.673
		6.67	1.651	1.607	116.678	222.234	1.207	1.164	1.632	1.867
		0.067	1.857	2.138	21.068	14.661	1.602	1.778	1.524	1.487
	IL-2	666.7	5436.838	5914.858	1964.568	4753.777	6.889	8.684	5.729	4.315
		6.67	54.83	48.016	85.826	67.234	4.564	6.903	3.435	2.234
		0.067	4.373	2.778	76.143	58.744	1.944	1.674	3.63	3.49
	IL-4	666.7	7.387	8.277	4.616	7.171	0.081	0.078	0.291	0.426
		6.67	0.132	0.123	1.906	1.786	0.043	0.027	0.031	0.018
		0.067	0.045	0.036	1.274	0.984	0.086	0.08	0.032	0.021
	IL-6	666.7	446.11	550.795	225.819	155.77	22.199	19.202	53.817	81.816
		6.67	9.425	5.32	214.786	280.205	4.788	4.001	6.216	3.311
		0.067	9.22	10.685	77.806	61.811	4.417	3.03	5.806	3.937
	IL-8	666.7	4940.504	3272.627	5501.218	2937.462	3058.645	3026.778	3403.905	3673.776
		6.67	701.713	794.044	4011.55	2136.535	326.481	369.514	703.74	930.95
		0.067	237.056	220.112	3135.889	1628.541	188.325	204.299	192.79	162.845
	TNF-α	666.7	2184.269	2215.865	1792.666	2223.487	45.286	34.798	215.239	402.911
		6.67	16.395	9.59	1046.923	1222.409	4.843	3.67	7.727	4.678
		0.067	3.931	3.58	395.545	306.79	3.18	2.363	3.509	2.096
Whole blood soluble phase	IFN-γ	666.7	1807.572	3383.398	108.301	189.587	17.123	32.121	17.662	23.183
		6.67	277.789	483.681	156.717	287.325	23.864	49.141	30.473	64.831
		0.067	30.415	64.565	35.413	51.353	35.39	76.684	23.571	47.588
	IL-10	666.7	151.868	134.067	0.728	0.644	0.282	0.111	0.199	0.095
		6.67	16.734	18.454	7.979	14.628	0.226	0.097	0.187	0.082
		0.067	0.511	0.467	0.587	0.503	0.455	0.484	0.229	0.126
	IL-12p70	666.7	2.87	2.824	0.186	0.17	0.158	0.053	0.108	0.048
		6.67	0.43	0.74	0.204	0.304	0.158	0.055	0.108	0.04
		0.067	0.17	0.098	0.152	0.054	0.164	0.08	0.154	0.065
	IL-13	666.7	57.713	42.969	1.163	0.608	0.95	0.603	0.648	0.461
		6.67	11.991	23.1	7.144	12.593	1.065	0.702	0.782	0.806
		0.067	1.617	0.931	1.252	0.724	1.03	0.712	0.972	0.868
	IL-1β	666.7	10.297	7.119	0.956	1.284	0.469	0.783	0.309	0.479
		6.67	1.166	1.275	1.239	1.666	0.513	0.797	0.307	0.447
		0.067	1.095	1.141	0.371	0.511	0.585	0.896	0.311	0.465
	IL-2	666.7	47.622	38.047	0.938	0.867	0.143	0.193	0.656	1.118
		6.67	199.012	472.861	8.202	13.297	0.358	0.361	0.195	0.208
		0.067	0.748	0.946	0.523	0.412	0.538	0.639	0.151	0.198
	IL-4	666.7	13.821	17.089	0.15	0.18	0.05	0.017	0.039	0.019
		6.67	3.56	7.792	0.984	1.931	0.051	0.029	0.051	0.046
		0.067	0.055	0.034	0.059	0.047	0.056	0.036	0.047	0.027
	IL-6	666.7	284.181	418.848	19.193	35.147	2.911	2.268	2.568	2.306
		6.67	54.707	120.339	14.523	26.854	2.671	2.184	2.767	2.303
		0.067	5.875	7.127	2.918	1.826	2.8	2.45	2.463	2.203
	IL-8	666.7	4318.48	472.415	121.008	67.597	82.332	60.874	70.439	33.786
		6.67	525.574	851.493	649.824	1117.967	94.092	94.449	86.633	79.225
		0.067	158.507	174.923	87.06	80.221	99.363	112.629	83.161	83.763
	TNF-α	666.7	47.385	49.282	5.307	5.611	2.32	2.224	2.375	1.921
		6.67	14.008	23.193	11.999	21.699	3.041	3.548	2.669	3.244
		0.067	3.289	4.045	3.032	2.712	2.875	3.372	2.664	3.189

Table S3. Cytokine release assay. Cytokine levels in supernatants after 24 hr culture of whole blood with antibodies. Numbers indicated cytokine concentrations (pg/ml). *only one of the donors demonstrated detectible cytokine levels, and value from the single donor is shown. For these data points, standard deviation is reported as ND (not determined).

	Cytokine	Antibody concentration (nM)	JTX-2011		Opdivo		JTX-2011/Opdivo		Herceptin®		anti-RSV-hG4		Herceptin®+anti-RSV-hG4		OKT3		Anc28.1	
			Mean	SD	Mean	SD	Mean	SD	Mean	SD	Mean	SD	Mean	SD	Mean	SD	Mean	SD
Whole blood soluble phase	IFN-γ	666.7	19.71	17.68	7.12	8.69	18.3	17.04	7.09	8.82	9.06	9.12	9.22	8.68	17.72	17.6	680.91	932.24
		66.7	18.83	15.78	8.7	8.82	19.96	17.89	8.94	8.62	10.54	8.92	9.02	8.86	15.26	13.82	525.26	516.89
	IL-10	666.7	0.07	0.06	0.1	0.02	0.09	0.07	0.1	0.08	0.15	0.07	0.18	0.04	0.51	0.42	90.32	86.87
		66.7	0.07	0.04	0.07	0.04	0.09	0.05	0.1	0.06	0.14	0.09	0.15	0.05	0.23	0.16	62.35	45.96
	IL-12p70	666.7	0.06*	ND	0.1*	ND	0.05	0	0.05*	ND	0.21	0.05	0.39	0.34	0.02*	ND	2.93	2.44
		66.7	<LLOD	ND	0.12	0.09	0.11*	ND	0.06*	ND	0.07	0.04	0.19	0.11	<LLOD	ND	1.35	0.41
	IL-13	666.7	1.26*	ND	1.19*	ND	0.71	0.96	0.99	0.94	1.24	1.18	1.3	1.22	1.14	0.71	36.85	50.33
		66.7	1.57*	ND	1.54*	ND	1.62*	ND	1	1	1.22	0.7	0.94	0.75	1.49*	ND	27.16	22.89
	IL-1β	666.7	<LLOD	ND	1.29*	ND	0.5*	ND	0.89*	ND	0.56	0.37	0.66	0.29	0.81*	ND	7.43	6.29
		66.7	<LLOD	ND	<LLOD	ND	<LLOD	ND	0.55	0.34	0.64*	ND	0.48*	ND	<LLOD	ND	4.43	2.2
	IL-2	666.7	0.12	0.02	0.11	0.03	0.21	0.11	0.12	0	0.14	0.05	0.16	0.02	2.33	2.54	35.64	30.95
		66.7	0.1	0.06	0.07*	ND	0.12	0.09	0.17	0.05	0.17	0.04	0.12	0.1	0.84	0.57	29.77	23.29
	IL-4	666.7	0.03	0.01	0.04	0.03	0.03	0.01	0.04	0.03	0.04	0.02	0.05	0.02	0.16	0.12	8.96	10.57
		66.7	0.02	0.01	0.05	0.02	0.03	0.02	0.04	0.01	0.05	0.02	0.05	0.02	0.06	0.05	6.16	4.83
	IL-6	666.7	1.13	0.46	1.7	1.99	1	0.44	0.83	0.37	1.18	0.52	1.29	0.58	1.81	1.42	157.95	198.29
		66.7	1	0.37	0.95	0.34	1.05	0.47	0.94	0.39	0.96	0.38	0.96	0.39	1.49	0.87	61.71	63.7
	IL-8	666.7	18.27	4.6	31.94	39.02	14.86	3.32	17.27	2.29	24.69	8.83	23.12	3.2	41.19	43.01	2014.22	2388.17
		66.7	21.41	9.08	19	4.84	18.01	4.62	18.58	5.73	19.06	6.37	17.69	3.34	33.11	16.49	1943.4	2047.56
	TNF-α	666.7	1.65	0.49	1.56	1.13	1.48	0.49	1.02	0.3	1.21	0.43	1.25	0.32	1.89	1.43	24.6	20.43
		66.7	1.62	0.67	1.17	0.5	1.66	0.71	1.15	0.48	1.21	0.36	1.2	0.27	1.63	0.99	22.55	15.16

Table S4. Cytokine release assay. Cytokine levels in supernatants after 24 hr culture of whole blood with antibodies. Numbers indicate cytokine concentrations in pg/mL. Antibodies were tested at either 66.67nM or 666.7nM concentrations. Means and SD from 6 donors are shown.

WB Soluble Phase		JTX-2011		Yervoy		JTX-2011/Yervoy		Herceptin		OKT3		Anc28.1	
		Mean	SD	Mean	SD	Mean	SD	Mean	SD	Mean	SD	Mean	SD
IFN-γ	666.7nM	26.4	21.5	6.7	6.4	29.3	24.7	8.1	9.5	2383.6	1197.3	70.6	112.5
	66.7nM	21.6	16.1	6.7	6.3	22.9	16.2	7.7	8.4	1860.8	983.8	62.8	94.2
IL-10	666.7nM	0.0	0.0	0.1	0.0	0.0	0.0	0.0	0.0	113.6	62.9	0.8	1.0
	66.7nM	0.0	0.0	0.0	0.0	0.0	0.0	0.1	0.0	116.7	75.2	1.1	1.9
IL-12p70	666.7nM	0.1	0.1	0.1	0.1	0.1	0.1	0.1	0.1	1.2	1.2	0.2	0.2
	66.7nM	0.1	0.1	0.0	0.1	0.1	0.1	0.1	0.1	1.0	1.1	0.1	0.1
IL-13	666.7nM	0.8	0.7	0.6	0.5	0.4	0.3	0.7	0.4	145.9	194.5	0.8	0.9
	66.7nM	0.7	0.7	0.5	0.3	0.4	0.1	0.9	0.7	134.9	165.2	1.0	1.2
IL-1β	666.7nM	0.2	0.2	0.2	0.3	0.4	0.4	0.2	0.1	10.7	14.5	0.4	0.3
	66.7nM	0.2	0.1	0.2	0.2	0.3	0.2	0.2	0.2	9.8	11.8	0.4	0.4
IL-2	666.7nM	0.1	0.0	0.1	0.0	0.1	0.0	0.1	0.0	124.3	127.1	0.3	0.3
	66.7nM	0.1	0.1	0.1	0.0	0.1	0.1	0.1	0.1	109.7	91.7	0.3	0.4
IL-4	666.7nM	0.0	0.0	0.0	0.0	0.0	0.0	0.0	0.0	39.9	66.9	0.1	0.1
	66.7nM	0.0	0.0	0.0	0.0	0.0	0.0	0.0	0.0	38.9	58.0	0.1	0.1
IL-6	666.7nM	1.1	0.9	1.6	2.6	1.2	1.2	0.5	0.3	194.2	126.1	2.1	1.8
	66.7nM	0.8	0.5	0.4	0.3	0.9	0.6	0.6	0.4	116.7	58.6	2.1	2.1
IL-8	666.7nM	56.1	27.0	70.8	99.4	68.6	38.2	48.4	38.4	4062.9	2109.4	103.0	66.1
	66.7nM	45.5	25.4	45.1	39.9	55.3	40.0	51.6	33.3	3754.2	2133.4	173.6	247.9
TNF-α	666.7nM	1.1	0.5	0.7	0.3	1.3	0.7	0.7	0.3	64.5	67.1	2.4	3.2
	66.7nM	1.0	0.5	0.7	0.3	1.1	0.5	0.7	0.3	47.7	38.7	2.6	3.3
